# The estimation of health state utility values in rare diseases: do the approaches in submissions for NICE technology appraisals reflect the existing literature? A scoping review

**DOI:** 10.1007/s10198-022-01541-y

**Published:** 2022-11-05

**Authors:** Michela Meregaglia, Elena Nicod, Michael Drummond

**Affiliations:** 1 Research Centre on Health and Social Care Management (CERGAS), SDA Bocconi School of Management, Milan, Italy; 2 Centre for Health Economics, University of York, York, UK

**Keywords:** Health state utility values, Rare diseases, National Institute for Health and Care Excellence, Technology appraisal, Scoping literature review

## Abstract

**Background:**

Rare diseases negatively impact patients’ quality of life, but the estimation of health state utility values (HSUVs) in research studies and cost–utility models for health technology assessment is challenging.

**Objectives:**

This study compared the methods for estimating the HSUVs included in manufacturers’ submissions of orphan drugs to the National Institute for Health and Care Excellence (NICE) with those of published studies addressing the same rare diseases to understand whether manufacturers fully exploited the existing literature in developing their economic models.

**Methods:**

All NICE Technology Appraisal (TA) and Highly Specialized Technologies (HST) guidance documents of non-cancer European Medicines Agency (EMA) orphan medicinal products were reviewed and compared with any published primary studies, retrieved via PubMed until November 2020, and estimating HSUVs for the same conditions addressed in manufacturers’ submissions.

**Results:**

We identified 22 NICE TA/HST appraisal reports addressing 19 different rare diseases. Sixteen reports presented original HSUVs estimated using EQ-5D or Health Utility Index (*n* = 12), direct methods (*n* = 2) or mapping (*n* = 2), while the other six included values obtained from the literature only. In parallel, we identified 111 published studies: 86.6% used preference-based measures (mainly EQ-5D, 60.7%), 12.5% direct techniques, and 2.7% mapping. The collection of values from non-patient populations (using ‘vignettes’) was more frequent in manufacturers’ submissions than in the literature (22.7% vs. 8.0%).

**Conclusions:**

The agreement on methodological choices between manufacturers’ submissions and published literature was only partial. More efforts should be made by manufacturers to accurately reflect the academic literature and its methodological recommendations in orphan drugs submissions.

**Supplementary Information:**

The online version contains supplementary material available at 10.1007/s10198-022-01541-y.

## Background

Rare diseases (RDs) are defined by the European Medicines Agency (EMA) as diseases with a prevalence of fewer than 5 in 10,000 people and affect around 30 million individuals in the European Union (https://ec.europa.eu). These diseases are often severe, life-threatening and/or disabling, characterized by an early onset. As such, they negatively affect patients’ and their carers’ quality of life (QoL) [1], which should be appropriately account for when assessing the benefit of a new medicine. This is usually done via consideration of patient-reported outcome (PRO) data and/or health state utility values (HSUVs).

According to the Food and Drug Administration (US), PROs are defined as ‘any report of the status of a patient’s health condition that comes directly from the patient, without interpretation of the patient’s response by a clinician or anyone else’, and a patient-reported outcome measure (PROM) as an instrument, usually a questionnaire, that captures PRO data from patients (or proxies) to measure disease impact or drug effects, in a clinical trial or other study [2, 3]. For some PROMs, known as preference-based measures, an algorithm has been developed that converts patient responses into HSUVs based on preferences for specific health states derived from the instrument’s dimensions. HSUVs are single numerical values expressing preference-weighted QoL for particular health states and scored on a scale ranging from 0 (for a state equivalent to ‘dead’) to 1 (for a state equivalent to ‘full health’) [4], although some instruments provide also negative values for health states considered worse than death [5]. Among their applications, they are used for calculating the quality-adjusted life year (QALY), which combines HSUVs and survival data in a single metric and is one of the most preferred outcome measures for health technology assessment (HTA) [4].

A number of methods have been developed to estimate HSUVs, including direct and indirect approaches (i.e., preference-based PROMs). The most common direct techniques include standard gamble (SG) and time trade-off (TTO), where respondents are asked to choose between life with a lower QoL, and life in ‘full health’ with a risk of immediate death (in SG) or a shorter length (in TTO) [6]. A simple approach is represented by the visual analogue scale (VAS), where individuals are asked to rate health by selecting a value on scale where 0 is the worst state they can imagine and 10 or 100 is the best [4]. In recent years, discrete choice experiments (DCEs) have become frequently used to generate HSUVs by asking individuals to choose between hypothetical health states to elicit their preferred health state and the relative weights for various attributes included within health states [7]. The use of an indirect approach through preference-based PROMs is increasingly common and encouraged by many HTA bodies. Six main preference-based generic measures for use in HTA were identified by a recent review [8]: the EuroQol 5-Dimension (EQ-5D), the Health Utility Index Mark 2 or Mark 3 (HUI2/3), the Short Form 6 Dimension (SF-6D), the Quality of Well-Being (QWB) scale, the 15D, and the Assessment of Quality of life (AQoL). In the United Kingdom, the National Institute for Health and Care Excellence (NICE) recommends the use of EQ-5D converted to QALYs to measure the added benefit of new drugs [9]. In detail, companies, academic groups and others preparing evidence submissions for NICE should use the EQ-5D-3L value set for reference-case analyses. If data were gathered using the more recent EQ‑5D‑5L descriptive system, utility values should be calculated by mapping the 5L data onto the 3L value set, until a new high-quality 5L value set for England becomes available [10]. A further approach that is gaining consensus in research and HTA, also accepted by NICE in the absence of EQ-5D data [11], is ‘mapping’ from non-preference-based measures onto preference-based ones (e.g., EQ-5D) using an algorithm previously developed.

The estimation of HSUVs in RDs, however, is challenging. This is because direct techniques may be too demanding for patients, who are often young children or mentally disabled, while generic preference-based instruments may not capture all the relevant symptoms and disabilities, and ‘vignettes’ that describe standard hypothetical health states to non-patient populations may not reflect the heterogeneous manifestations of these diseases [12]. Similarly, the use of mapping in RDs is characterized by difficulties in recruiting sufficiently large samples needed to conduct the regression analysis to derive the algorithm, the limited overlap between the domains included in the disease-specific and generic PROMs, and the poor applicability of algorithms developed for non-rare conditions [13]. Disease-specific preference-based PROMs, which are more sensitive measures, exist for only few RDs (e.g., the Amyotrophic Lateral Sclerosis Utility Index, ALSUI) [14]. Caregiver QoL is increasingly considered in HTA, and several carer-specific preference-based instruments have been developed (e.g., ASCOT-Carer and CarerQol-7D) [15, 16]. However, their use and consideration in RDs remain limited [17, 18], even though most diseases affect young children impacting the QoL of those caring for them.

Work Package 10 of the EU Horizon 2020 project IMPACT-HTA (www.impact-hta.eu) aimed to investigate and develop guidance on use of PRO data and HSUVs in RD treatments for HTA. Results suggested PROs and HSUVs sometimes fail to demonstrate change in symptomatology or capture dimensions that really matter to patients, and their estimates are often uncertain and/or of poor quality due to insufficient evidence. The research also pointed to the likely impact of the nature of RDs on the methodological limitations identified, e.g., collecting PROs from paediatric, cognitively impaired, or heterogeneous populations [12, 19].

When preparing their HTA submissions, manufacturers usually review the available literature to identify and derive the parameters for their economic evaluations and/or to learn about methods, such as existing techniques to estimate HSUVs. Given the poor quality of the QoL evidence included in many HTA submissions [18], the question is whether manufacturers are making full use of the available literature, or whether the poor quality might be a consequence of the intrinsic limitations in applying the existing approaches to PRO and HSUV data generation in RDs. In order to answer this question, this paper aimed to further examine the approaches used to derive the HSUVs used in HTA submissions and compare them with the corresponding methodologies and recommendations arising from the literature. This was done by reviewing and comparing the methods used by manufacturers to derive HSUVs in NICE’s appraisal reports of orphan drugs with all published studies addressing the same RDs to understand whether manufacturers fully exploited the existing literature in developing their economic models and derive some related methodological learnings.

## Methods

All treatments with an EMA orphan designation and appraised by NICE within their Technology Appraisal (TA) and Highly Specialized Technologies (HST) programmes until June 2020 were included in the study. Specialized or selected high-cost low-volume treatments for very rare conditions are typically evaluated within the HST procedure, while all other treatments undergo the TA process [20]. We excluded appraisal reports for cancer indications given that QALYs are more likely to be driven by survival rather than QoL increases, and any reports for conditions that are not included in the ORPHANET list of RDs [21].

The publicly available appraisal reports were downloaded from NICE’s website (https://www.nice.org.uk/). These reports include a summary of the evidence submitted by the manufacturer, a review of the manufacturer’s submission by an independent Evidence Review Group (ERG), and the Appraisal Committee’s appraisal of the evidence and final decision. The following information was extracted about the manufacturer’s approach(es) embraced to derive the HSUVs used in the economic model(s): type of technique(s) used, number and type of respondents, and literature sources consulted. Subsequent comments, criticisms, and suggestions made by the ERG and/or Committee relating to the techniques used and HSUVs results were also extracted and summarized in order to gain a better understanding of NICE’s opinion of these approaches or of what they would consider reasonable to obtain HSUVs.

We then performed a scoping review of published studies following the Preferred Reporting Items for Systematic reviews and Meta-Analyses extension for Scoping Reviews (PRISMA-ScR) checklist [22]. The scoping review was considered appropriate to synthesize the literature as this approach is suggested to map evidence and investigate how research is conducted on a certain topic (i.e., in this study, the estimation of HSUVs in rare diseases) [22, 23]. The aim was to retrieve all primary studies estimating HSUVs in the same indications addressed by the sample of NICE reports identified. An example of the research string used in PubMed is reported below for one NICE appraisal assessing Burosumab for X-linked hypophosphatemia (HST8 [24]):

*((Burosumab)[Title/Abstract] OR (X-linked hypophosphatemia)[Title/Abstract]) AND ((health state utility values)[Title/Abstract] OR (utility values)[Title/Abstract] OR (health utilities)[Title/Abstract] OR (preference weights)[Title/Abstract] OR (index values)[Title/Abstract] OR QALYs[Title/Abstract] OR (cost-utility)[Title/Abstract] OR EQ-5D[Title/Abstract] OR EuroQol[Title/Abstract] OR HUI[Title/Abstract] OR (Health Utility Index)[Title/Abstract] OR QWB[Title/Abstract] OR SF-6D[Title/Abstract] OR 15D [Title/Abstract]).*

The same string was applied to every drug-disease combination addressed by the NICE reports identified; drugs for the same indication were included in a single string (the full search strategy is reported in Supplementary File 1). The electronic searches were conducted until November 2020. The ScHARRHUD database (https://www.scharrhud.org/) holding bibliographic details of studies reporting HSUVs was also searched. All search results were extracted into an Excel spreadsheet and duplicates removed. Titles and abstracts were screened by two independent reviewers and records excluded if they did not meet the inclusion criteria; full-text papers were retrieved in case of doubtful results. Any disagreement was solved by discussion until a consensus was reached.

The studies deemed eligible for inclusion were those presenting an original approach for estimating HSUVs in any of the conditions of interest, i.e., we excluded those using estimates from previous studies already in the literature. Studies addressing multiple conditions (e.g., post lung transplantation patients) were included provided that at least one subsample of observations was related to the condition of interest (e.g., cystic fibrosis). Studies of any design (i.e., cross-sectional surveys, cohort studies, clinical trials, and cost–utility models) could be included in the review, provided that they showed an original approach to deriving HSUVs. Studies in languages other than English, conference abstracts and study protocols were excluded. Literature reviews were excluded but their reference lists were manually checked in order to identify any additional original studies not captured by the online searches. Similarly, economic evaluations using the published literature to obtain utility parameters for their analyses were excluded, but their reference lists were checked to avoid missing any relevant publications.

For each study, we extracted detailed information regarding the study characteristics (i.e., study design, setting, country, type and number of participants), the method(s) adopted to estimate HSUVs (e.g., direct approaches, such as SG or TTO, indirect approaches using preference-based instruments, such as EQ-5D or mapping), actual HSUVs estimates for the study sample and relevant subgroups, and authors’ methodological considerations regarding the approaches used to estimate HSUVs (if reported). The data extraction form (in Excel) was piloted in parallel by two reviewers on a sample of ten studies, and subsequently refined and completed independently by the first author. The study characteristics were tabulated and subsequently summarized through descriptive statistics (i.e., frequency distribution).

Then, the methods used by manufacturers to derive HSUVs, as reported in the NICE documents reviewed, were compared with those used in the published literature for each indication considered. Specifically, we aimed to understand whether manufacturers relied on the published studies (in the public domain at least one year before the NICE appraisal date) to retrieve HSUVs, or to replicate the techniques adopted to derive HSUVs. Otherwise, we sought to understand if divergences from the existing literature was motivated by the limitations highlighted by study authors’ around the technique(s) adopted and the results obtained. In addition, based on these authors’ methodological considerations, we summarized the key learnings that are important to account for when using each of the available methods to derive HSUVs in individual RDs, and whether the manufacturer’s approach could have better reflected any methodological advice in the existing literature.

## Results

### Synthesis of NICE technology appraisals

**Fig. 1:**
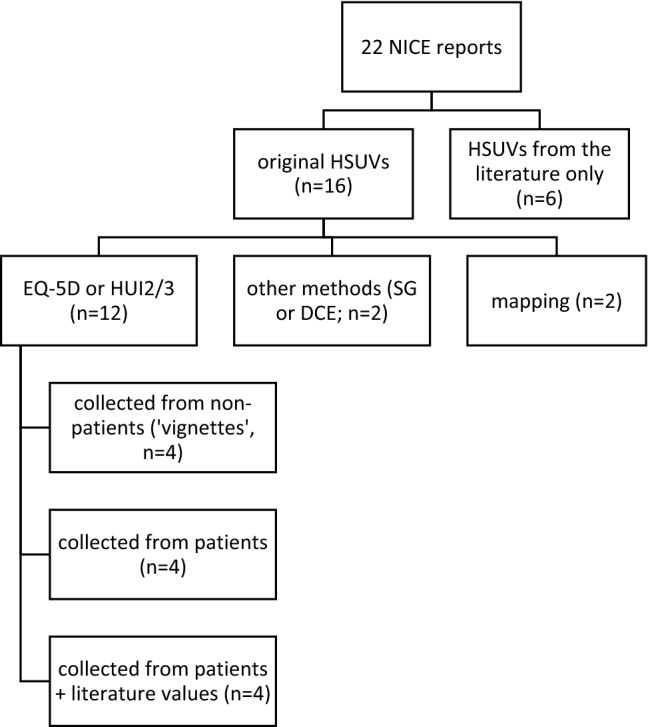
Synthesis of methods used by manufacturers to obtain HSUVs (as reported in NICE TA/HST guidance documents)

Of the 48 appraisal reports identified from the NICE website, we excluded 24 for cancer indications and two additional reports for Crohn’s and cytomegalovirus disease, since these conditions are not included in the ORPHANET list of RDs. The final sample included 22 TA/HST reports [24–45] for 19 different indications (one indication could have several treatments appraised), published between 2012 and 2019 (Table 1). Across these 22 manufacturer submissions, 16 (72.7%) derived their own HSUVs. Of these, 12 presented original data collection using a preference-based instrument (i.e., EQ-5D in 10 reports, HUI2 in one report, and HUI3 together with EQ-5D in another one). In eight of these (HST1, HST10, TA266, TA398, HST2, TA431, TA491, TA379), questionnaires were filled in by patients within a clinical study, while in the remaining four (HST6, HST8, HST11, HST12) questionnaires were administered to clinical experts to value hypothetical health state descriptions (i.e., ‘vignettes’). In two reports (TA276 and HST5), HSUVs were derived by mapping non-preference-based measures (Cystic Fibrosis Questionnaire, CFQ and Short Form-36, SF-36) onto EQ-5D. In one report (HST4), HSUVs were obtained through a discrete choice experiment (DCE), and in another (TA467) through a SG exercise, both performed with the general public. Finally, six reports (HST3, HST7, HST9, TA443, TA588, TA606) did not perform any empirical study, and relied only on literature values and/or expert opinion to obtain HSUVs (Fig. 1).

**Table 1:** Estimation of HSUVs in RDs: a review of NICE TA/HST guidance documents

Report code (ref.)	Drug	Manufacturer’s approach	NICE/ERG’s notes/recommendations
Report date	Disease		
HST6 [25]	Asfotase alfa	Mean utility values included in the company’s economic model were estimated by 9 clinical experts who completed the EQ-5D-5L for vignettes for each health state based on the 6MWT severity levels	The ERG felt that deriving HSUVs from clinical experts rather than from clinical studies is a limitation. Moreover, the 6MWT does not capture all the symptoms of hypophosphatasia and the EQ-5D domains, although clinicians may have considered these when providing HSUVs for the illustrative vignettes. However, the HSUVs obtained by the experts seem reasonable.
2/8/2017	Paediatric-onset hypophosphatasia		
HST1 [26]	Eculizumab	EQ-5D was collected from 37 patients within two clinical studies (C08-002A/B and C08-003A/B)	None
28/1/2015	Atypical haemolytic uremic syndrome		
HST4 [27]	Migalastat	Complication-related disutilities were derived from a group with Fabry diseases. Infusion-related disutilities were derived from a DCE performed with 506 people from the UK general population.	The ERG noted uncertainty about the comparability of infusion disutilities with those of disease complications given the differences in the methods used for estimation. The ERG did a scenario analysis with utilities derived from alternative sources and reduced infusion-related disutilities.
22/2/2017	Fabry Disease		
HST9 [28]	Inotersen	Original approach: published literature (Stewart 2017, which reports EQ-5D utilities using a Brazilian value set). Revised approach: using one or two EQ-5D health states in which the values from the Brazilian data were closest to the mean disease stage values for patients in the preferred THAOS registry (i.e., a global registry owned by another company).	The ERG argued that using EQ-5D values based on Brazilian general population preferences was questionable because there are important differences in preferences for health states between the UK and Brazilian population. The revised approach, although not optimal, was acceptable for decision-making.
22/5/2019	Hereditary transthyretin amyloidosis		
HST10 [29]	Patisiran	The company used the EQ-5D-5L utility values collected in APOLLO trial mapped to EQ-5D-3L (using Van Hout 2012) for a regression model relating HRQoL to PND scores and the interaction between time and treatment	The ERG considered the regression to be unreliable because it excluded important parameters (e.g., cardiac involvement) and included the interaction between time and treatment without the main terms (i.e., time and treatment).
14/8/2019	Hereditary transthyretin amyloidosis		
HST11 [30]	Voretigene neparvovec	Vignettes were developed for each health state using clinicians and patient input. The company then asked six clinicians to complete HUI3 and EQ-5D for each health state in the economic model. The company preferred to use HUI3 because it contains a visual component.	The committee noted that the company’s methods have several serious limitations: (1) small number of clinicians taking part in the vignette study; (2) ophthalmologists may focus on issues related to vision loss, which may have underestimated the overall HRQoL. The ERG suggested using utilities from Rentz 2014, a general public time trade-off study that looked at 8 health states with varying degree of vision problems defined by the NEI VFQ-25 items. The committee considered that neither source of data was sufficiently robust but the HSUVs in the models are likely to fall between the values from Rentz et al. and the EQ-5D company values.
9/10/2019	Inherited retinal dystrophies		
TA266 [31]	Mannitol dry powder	HUI2 was collected from patients in a clinical trial (DPM-CF-302); literature values were also included for lung transplantation and pulmonary exacerbations.	The committee was concerned by the use of HUI2 instead of EQ-5D that is the preferred measure by NICE and was not convinced that HRQoL of patients had been valued with any certainty.
28/11/2012	Cystic fibrosis		
TA398 [32]	Lumacaftor–ivacaftor	A multivariate mixed-model repeated measures regression analysis was used to model the relationship between EQ-5D utility values, lung function (ppFEV_1_) and pulmonary exacerbations reported in TRAFFIC and TRANSPORT trials. Utility values for lung transplants were taken from Whiting 2014.	The committee appreciated that the company had included EQ-5D data, as preferred by NICE. There was no evidence to suggest that the EQ-5D was inappropriate and it generally captured the effects of having cystic fibrosis and its treatment.
27/07/2016	Cystic fibrosis		
TA276 [33]	Colistimethate sodium and tobramycin dry powders for inhalation	CFQ data collected in a clinical trial were mapped to EQ-5D using published coefficients (Eidt-Koch 2009)	The Assessment Group highlighted the shortcomings in the use of the mapping approach to produce HSUVs. The ERG developed a de novo model deriving HSUVs from the Bradley (2010) study.
27/3/2013	Pseudomonas lung infection in cystic fibrosis		
HST2 [34]	Elosulfase alfa	Original approach: HSUVs were based on the general population (asymptomatic state), an observational study of the natural history of MPS IVa using EQ-5D-5L, and cross-sectional surveys of people with the conditions and their families. Revised approach: HSUVs were obtained from the literature.	The committee noted that the effect of the condition on HRQoL had been assessed using EQ-5D-5L in a natural history study, while the clinical trials on elosulfase alfa collected only limited evidence on HRQoL and did not collect EQ-5D. Therefore, although it was reasonable to include a utility increment with elosulfase alfa, the existing evidence did not allow this benefit to be estimated robustly.
16/12/2015	Mucopolysaccharidosis type IVa		
HST8 [24]	Burosumab	The company conducted a utility study in which vignettes describing the modelled health states were developed. The vignettes were valued using EQ-5D-5L by 6 clinical experts. An additional value was inferred for the healed health state.	The committed noted that the utilities were scored by clinicians not patients, and were not taken directly from trials, which were limitations of the data. It concluded that the utility values were uncertain but, in the absence of an alternative, were acceptable for decision-making.
10/10/2018	X-linked hypophosphatemia		
HST7 [35]	Strimvelis	Quality of life data collected in the STRIMVELIS clinical trials were too limited to be included in the model. The company instead used utilities from the literature. No disutility was considered for people having intravenous immunoglobulin (IVIG) or for those who had severe infections. The impact of these assumptions was explored in sensitivity and scenario analyses.	The ERG preferred to incorporate the company's scenario analysis in which a utility weight was applied to people who had IVIG. The committee concluded that, because the ERG's preferred assumptions were based on available evidence, they were preferable for decision-making.
7/2/2018	Adenosine deaminase deficiency-severe combined immunodeficiency		
TA588 [36]	Nusinersen	The company generated patient utilities from the clinical advisers. The carer-related utilities used by the company assumed that the best health state was associated with general population utility, and the worst health state was the average carer utility from a literature source.	The committee recognised that identifying robust utility values in babies and young children is exceptionally challenging. The ERG considered the company’s approach as the most appropriate. However, it noted that the utility estimates should be considered cautiously because they are not based on formal elicitation methods, may be different if other clinicians valued the health states and may not accurately reflect the view of people with SMA or their carers. Moreover, the estimates of carer utilities used in the model should be treated with caution because most were driven by assumptions rather than by evidence.
24/7/2019	Spinal muscular atrophy		
TA431 [37]	Mepolizumab	EQ-5D was collected in DREAM trial	The committee noted that the company did not adjust utilities by age because DREAM showed there was no difference between age and utility. The committee considered the ERG's comment that DREAM was not powered to detect age-dependent utilities and noted that there were fewer patients underpinning the data for utilities in older people. The committee concluded that it preferred the ERG's base case, which applied age-adjusted utilities.
25/1/2017	Severe refractory eosinophilic asthma		
HST5 [38]	Eliglustat	The SF-36 collected in the GD-DS3 score study was mapped to EQ-5D using a published algorithm. The utility increment (0.12) of oral therapy over infusion therapy was taken from a vignette study	The ERG agreed that the GD-DS3 score study provided the most complete set of utility values. It also agreed that oral therapy would provide a clear quality-of-life benefit but questioned the extent of the benefit assumed by the company, even though this was based on a vignette study, and proposed the alternative utility increment of 0.5. The committee concluded that, although the true value was uncertain, the alternative value used by the ERG was more appropriate.
28/6/2017	Gaucher disease (type 1)		
TA467 [39]	Holoclar	The value of the utility decrement for disfigurement used in the company’s model was taken from a bespoke standard gamble (SG) stated preference exercise in 520 UK participants who were presented with various clinical scenarios describing moderate to severe LSCD, including an image of a patient’s eye with this condition. The estimated utility decrement for disfigurement was consistent with the opinion of clinical experts.	The committee noted that the utility values used in the company’s model were far lower than any used in previous appraisals for eye treatments, noting that ERG had used alternative values. For disfigurement, it used a decrement of 0.140 (rather than the company's assumption of 0.318), using cataracts as a proxy. The committee recognised that cataract disutilities were used as a proxy for disfigurement, and although uncertainty remained in the utilities, the ERG's values were a more realistic reflection of the impact on HRQoL.
16/8/2017	Limbal stem cell deficiency after eye burns		
TA606 [40]	Lanadelumab	The company used utility values from Nordenfelt 2014, which is a Swedish study that included EQ-5D-5L values for both the attack-free and the attack health states. The company explained that the EQ-5D-5L values collected in the HELP-03 trial were limited and could not be used in the model.	The committee concluded that the company's preferred utility values that included a benefit for lanadelumab subcutaneous administration were acceptable for decision-making
16/10/2019	Hereditary angioedema		
TA443 [41]	Obeticholic acid	HRQoL data were not collected in POISE trial so the company used utility values from published literature (Younossi 2001 and Wright 2006, used in NICE's technology appraisal guidance on sofosbuvir for treating chronic hepatitis C)	The committee acknowledged the uncertainty associated with the utility values but accepted that they had been derived from published sources
26/4/2017	Primary biliary cholangitis		
TA491 [42]	Ibrutinib	EQ-5D-5L data were collected in the RESONATE CLL trial. The utility decrements associated with progression and adverse events were based on published literature (Beusterien 2010, Tolley 2013).	Clinical advisors to the ERG noted that given the lack of HRQoL data available for patients with WM, the use of utilities from a CLL study by proxy may be reasonable
22/11/2017	Waldenstrom’s macroglobulinaemia		
TA379 [43]	Nintedanib	The company assigned utility values to each health state in the model using EQ-5D data collected in the INPULSIS trials	The committee approved of the company using trial-based EQ-5D data to estimate HSUVs
27/1/2016	Idiopathic pulmonary fibrosis		
HST3 [44]	Ataluren	The company model included HRQoL data from the literature (Landfeldt et al. 2014) to inform the utility values for patients and carers	The committee concluded that it is imperative that its future review includes carer utility data
20/7/2016	Duchenne muscular dystrophy		
HST12 [45]	Cerliponase alfa	The company commissioned a utility study in which vignettes describing health states for both cerliponase alfa and standard care were developed. The vignettes were validated by a clinical expert and sent to 8 clinical experts who completed the EQ-5D-5L questionnaire as a proxy for patients. These were mapped to the EQ-5D-3L before being applied in the model. The company also included disutilities for carers and siblings.	The committee was concerned about the robustness of the vignettes used to elicit these utility values. It noted that they contained additional disease elements that had an unclear association with the motor and language scale that defined the health states. However, it concluded that, in the absence of further evidence, it would consider EQ-5D-3L values estimated from the utility study using vignettes. The committee was satisfied with the principle of including disutility values for carers and siblings but agreed with the ERG that applying them for the whole 95-year time horizon was unrealistic given the life expectancy of parents, and also because disutility may change as siblings grow up and move on.
27/11/2019	Neuronal ceroid lipofuscinosis type 2		

*6MWT* 6-min walk test, *CFQ* cystic fibrosis questionnaire, *CLL* chronic lymphocytic leukaemia, *EQ-5D* EuroQol-5 Dimension, *EQ-5D-Y* EuroQol-5 Dimension-Youth, *ERG* Evidence Review Group, *HRQoL* health-related quality of life, *HST* highly specialized technology, *HSUVs* health state utility values, *HUI* Health Utility Index, *LSCD* limbal stem cell deficiency, *SMA* spinal muscular atrophy, *NEI VFQ* National Eye Institute Visual Function Questionnaire, *NICE* National Institute for Health and Care Excellence, *PND* polyneuropathy disability, *TA* technology appraisal

In total, the use of ‘vignettes’ was reported in five cases (22.7%), of which four (HST6, HST8, HST11, HST12) using EQ-5D or HUI2/3, and one (TA467) SG, and ten reports (HST9, TA266, TA398, HST2, HST7, TA588, TA606, TA443, TA491, HST3) referred to published studies to obtain some or all HSUVs for their analyses. Cumulatively, 15 cases (68.2%) reported EQ-5D utilities obtained with different approaches: seven (HST1, HST2, HST10, TA379, TA398, TA431, TA491) collecting data directly from patients, four (HST6, HST8, HST11, HST12) using ‘vignettes’ to be valued by clinical experts, two (HST9, TA606) referring to published studies, and other two (HST5, TA276) using ‘mapping’.

The Committees and/or the ERG appraised six cases positively, where the HSUVs were derived either from EQ-5D data collected within a trial, or from different approaches considered to be reasonable (HST1, TA398, TA606, TA443, TA491, TA379). In 10 cases (HST2, HST3, HST5, HST6, HST8, HST9, HST11, HST12, TA431, TA588), the Committees highlighted several limitations in the approach used by the manufacturer (i.e., surveys of clinical experts instead of patients, small samples, EQ-5D value sets from other countries, data collected within observational studies instead of trials, vignettes with unclear disease elements) but recognized that these could be acceptable with some adjustments and/or cautious consideration of results. In the remaining six reports (HST4, HST10, TA266, TA276, HST7, TA467), the Committees were more sceptical about the approach adopted, considering that it might yield unrealistic HSUVs estimates. Consequently, they often preferred to rely on alternative de novo approaches developed by the ERG.

### Literature search results

**Fig. 2:**
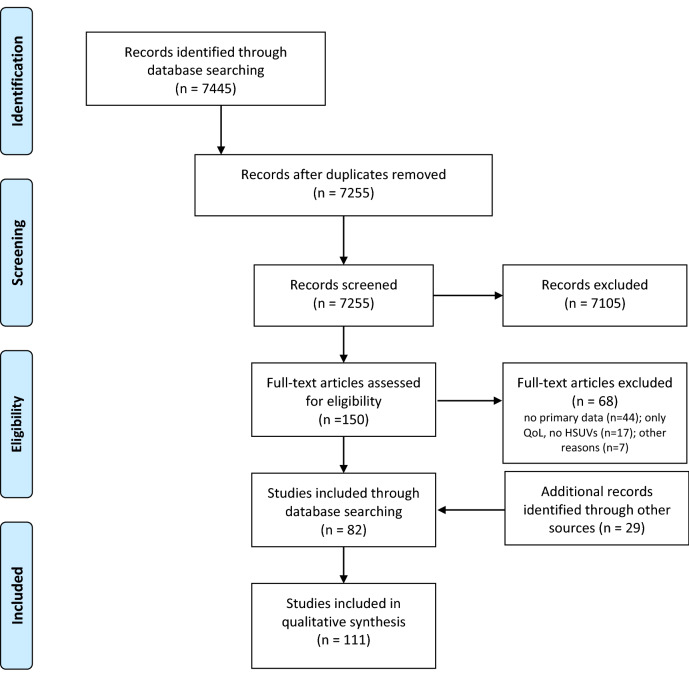
PRISMA 2009 Flow Diagram showing study selection

In total, the search strategy in PubMed identified 7445 articles. After removing 190 duplicates, 7255 records were scanned for title/abstract and 7105 were excluded in this first phase. Subsequently, 150 full-text articles were retrieved, and a further 68 records were excluded for not complying with the inclusion criteria. The two main reasons for exclusion were that the study evaluated QoL but did not estimate HSUVs, or that the study used HSUVs from the literature. Accordingly, 82 studies were selected, plus 29 studies identified through manual searches, resulting in a total of 111 studies included in the review [46–156]. No additional articles were obtained from the ScHARRHUD database (Fig. 2).

### Synthesis of published studies

The 111 studies estimated HSUVs in the same RDs addressed by the NICE reports (Tables 2, 3). One study [152] was reported twice since it addressed two different conditions, thus leading to the consideration of 112 different studies. At least one study was identified for each RD addressed in the NICE reports, with the exceptions of paediatric-onset hypophosphatasia and neuronal ceroid lipofuscinosis. The highest number of publications were retrieved for cystic fibrosis (*n* = 36, 32.1%), idiopathic pulmonary fibrosis (*n* = 16, 14.3%), and Duchenne muscular dystrophy (DMD, *n* = 10, 8.9%).

**Table 2:** Estimation of HSUVs in RDs addressed by NICE TA/HST guidance documents: results from the literature review

Report code (date) Drug/disease	First author (year) [ref.]		Study design/main purpose	Sample size (country)	Technique(s) to derive HSUVs	Main findings (HSUVs estimates)	Authors’ methodological considerations
HST1 (28/1/2015) Eculizumab/ Atypical haemolytic uremic syndrome	Fakhouri (2016) [76]		Open-label single-arm phase 2 trial of eculizumab to assess its efficacy in inhibiting complement-mediated thrombotic microangiopathy (TMA)	38 patients of 18 years or older with AHUS (US)	EQ-5D (US value set)	Mean change from baseline was significant (*p* < 0.001)	None
	Legendre (2013) [103]		Two prospective phase 2 trials of eculizumab to assess the changes in the platelet count (trial 1) and TMA event-free status (trial 2)	35 patients of 12 years or older with AHUS (Europe and US)	EQ-5D	Mean increase in EQ-5D score at week 26: trial 1: 0.32 (95% CI, 0.24–0.39; *p* < 0.001); trial 2: 0.10 (95% CI, 0.05–0.15; *p* < 0.001)	None
	Licht (2015) [105]		Extension of two phase 2 studies of eculizumab (Legendre 2013) to assess outcomes after 2 years	32 patients of 12 years or older with AHUS (Europe and US)	EQ-5D	Baseline median EQ-5D (range): trial 1: 0.8 (0.3–1.0); trial 2: 0.9 (0.2–1.0). Eculizumab significantly improved the EQ-5D score over 2 years (*p* < 0.05 compared with baseline)	None
HST4 (22/2/2017) Migalastat/Fabry Disease	Arends (2018) [51]		Retrospective cohort study to assess HRQoL	286 patients (NL and UK)	EQ-5D-3L (Dutch and UK preference weights)	Mean EQ-5D index score: 0.77 (± 0.26)	The EQ-5D offers three answer options per domain, limiting the detection of small changes in health
	Beck (2004) [54]		Cohort study to monitor the long-term effects of ERT with agalsidase alfa on renal function, heart size, pain and QoL	545 patients (11 European countries)	EQ-5D-3L (UK preference weights)	There was a significant improvement in the EQ-5D utility score during the first year of treatment with agalsidase alfa (*p* < 0.05)	None
HST4 (22/2/2017) Migalastat/Fabry Disease	Hoffmann (2005) [88]		Prospective study to evaluate pain and its influence on QoL in patients with Fabry disease receiving ERT with agalsidase alfa using data from the Fabry Outcome Survey (FOS)	314 patients with Fabry disease receiving ERT with agalsidase alfa and registered on the FOS European database	EQ-5D-3L	The mean EQ-5D utility score prior to ERT was 0.66 (0.32, *n* = 120). After 12 months of treatment with agalsidase alfa, this had improved to 0.74 (0.26; *n* = 59; *p* < 0.05); this improvement was maintained after 2 years (*n* = 28)	The EQ-5D has some limitations. It was established to detect and to measure changes in HRQoL reported by the patient; therefore, improvement of HRQoL does not imply improvement in an individual’s physical health state, but an improvement in the patient’s perception of their health state.
	Mehta (2009) [114]		Analysis of the Fabry Outcome Survey (FOS) observational database to assess cardiac mass and function, renal function, pain and QoL	181 adult patients from 19 countries	EQ-5D	HRQoL, measured by mean (SD) deviation scores from normal EQ-5D values, improved significantly, from –0.24 (0.3) at baseline to –0.17 (0.3) after 5 years (*p* = 0.0483)	None
	Miners (2002) [115]		Baseline data of a trial involving replacement therapy with a-galactosidase to assess HRQoL	38 males with AFD (UK)	EQ-5D	Mean (SD) EQ-5D utility: 0.56 (0.35). Mean (SD) EQ-VAS: 24.3 (31.1)	None
	Ramaswami (2012) [128]		Use of registry (Fabry Outcome Survey, FOS) data to validate FPHPQ	87 children (4–18 years) from eight countries	EQ-5D	EQ-5D utility (mean ± SD): 1.00 ± 0.00. EQ-VAS: 1.11 ± 0.31	Because Fabry Disease is a rare disease, it was necessary to pool data of multiple languages to be able to include patients with a wide range of disease severity and to ensure a sufficient sample size for data analysis
HST4 (22/2/2017) Migalastat/Fabry Disease	Rombach (2013) [131]		Cost-effectiveness analysis of ERT compared to standard medical care	142 patients aged 5–78 years (Netherlands)	EQ-5D-3L (UK preference weights)	Mean (95% CI) EQ-5D utility: asymptomatic: 0.874 (0.804–0.934); acroparesthesia (symptomatic): 0.762 (0.699–0.822); single complication: 0.744 (0.658–0.821); multiple complications: 0.584 (0.378–0.790); total: 0.772 (0.729–0.815)	None
	Tsuboi (2012) [144]		Prospective observational study (6-month intervals assessments) to assess the feasibility of switching patients on agalsidase beta treatment to agalsidase alfa instead in terms of renal function, cardiac mass, pain, QoL, and tolerability/safety	11 patients (Japan) in whom the treatment was switch from agalsidase beta to agalsidase alfa	EQ-5D-3L	The EQ-5D index score confirmed the stabilization of Fabry disease relative to pre-switch values	None
	Wyatt (2012) [152]		Cost-effectiveness analysis of ERT for lysosomal storage disorders (LSDs)	311 patients (UK)	EQ-5D and SF-36 derived (SF-6D) (UK preference weights)	None	None
HST9 (22/5/2019) Inotersen/Hereditary transthyretin amyloidosis	Inês (2020) [89]		Prospective cohort study to assess HRQoL	621 asymptomatic carriers and 733 symptomatic patients (Portugal)	EQ-5D-3L (Portuguese value set)	Among patients, the utility value is estimated to be 0.51 (0.021), a decrement of 0.27 as compared with general population utility. No differences on utility were found between carriers and general population (*p* = 0.209)	EQ-5D-3L is a valid instrument in assessing HRQoL among hATTR-PN patients. Difficulties remain due to disease rarity and small samples
HST10 (14/8/2019) Patisiran/Hereditary transthyretin amyloidosis	Obici (2020) [119]		Randomized placebo-controlled trial (APOLLO) of patisiran to describe its impact on QoL	225 patients (*n* = 148, patisiran; *n* = 77, placebo) from 21 countries	EQ-5D-5L	EQ-5D score: baseline: 0.6 (0.2) in both groups; at 18 months: + 0.2 in patisiran). EQ-VAS: baseline: 55.7 (20.0) for patisiran and 54.6 (18.0) for placebo; at 18 months: + 9.5 for patisiran	QoL tools used in APOLLO were appropriate measures for this disease
	Wixner (2014) [151]		Use of baseline data from a global, multicentre, longitudinal, observational survey (Transthyretin Amyloidosis Outcomes Survey, THAOS) to explore the prevalence and distribution of gastrointestinal manifestations in transthyretin amyloidosis and to evaluate their impact on HRQoL	1579 patients from seventeen countries	EQ-5D (US value set)	Gastrointestinal symptoms were significant negative predictors of the EQ-5D index score	None
HST11 (9/10/2019) Voretigene/Inherited retinal dystrophies	Brown (1999) [61]		Cross-sectional study to determine the relationship of visual activity loss to QoL	325 patients (included 1 with x-linked retinoschisis) in the US	Standard gamble and time trade-off	Utilities associated with the better-seeing eye [mean (SD)]: SG: 0.85 (0.21); TTO: 0.77 (0.23) (*p* < 0.001)	It is the opinion of the author that patients understand the TTO concept more readily than the SG concept. Additionally, as was the case in this study, evidence has shown that the SG method overestimates risk aversion.
HST11 (9/10/2019) Voretigene/Inherited retinal dystrophies	Davison (2017) [70]		Prospective cohort study to explore the feasibility of delivering standardized genomic care and of using selected measures to quantify its impact on patients	98 patients with IRD and receiving standardized multidisciplinary care (UK)	EQ-5D-3L (UK preference weights)	EQ-5D (complete case; *n* = 51): baseline: 0.747; 1 month: 0.744; 3 months: 0.794. EQ-5D (multiple imputation; *n* = 98): baseline: 0.778; 1 month: 0.776; 3 months: 0.810	Because the EQ-5D displayed considerable ceiling effects, further empirical work is needed to determine whether it is suitable for use in populations with genetic eye conditions. However, having the data enables comparisons across populations and health conditions.
	Lloyd (2019) [107]		Cross-sectional study to develop health state descriptions of RPE65-mediated IRD and to estimate associated patient utilities	6 ophthalmologists (US)	(1) Development of vignettes through background materials, feedback from an expert advisory board meeting, and interviews with clinical specialists, patients, and caregivers. (2) Proxy evaluation of vignettes by retina specialists with additional expertise in IRDs by using EQ-5D-5L (mapping to 3L UK value set) and HUI3	The EQ-5D-5L weights ranged from 0.709 for moderate vision loss to 0.152 for hand motion to no light perception (NLP). The HUI3 weights ranged from 0.519 to −0.039, respectively. A decline was seen on both measures, and the degree of decline from moderate vision loss to NLP was identical on both (− 0.56)	Given the ultra-rare nature of RPE65-mediated IRD, it was not feasible to recruit a patient sample and collect HRQL data prospectively. The qualitative picture that informed the vignettes supports the face validity of the utility findings. Clinicians were favoured over the general public for rating vignettes because the experience of severe vision loss may be difficult for the public to imagine. It also allowed describing some specific clinical information that may not be readily understood by the general public. The utility values are low, especially for the more severe states, and the qualitative work supported these scores.
TA266 (28/11/2012) Mannitol dry powder/cystic fibrosis	Acaster (2015) [48]		Cross-sectional survey to develop a mapping algorithm to estimate EQ-5D utility values from CFQ-R data	401 patients with CF (UK)	Mapping from CFQ-R to EQ-5D-3L (UK preference weights)	The mean EQ-5D score was 0.67 (SD: 0.28), ranging from –0.35 to 1	There was a tendency of over prediction in all models for observed values of EQ-5D lower than 0.3, and to a lesser extent, under prediction above observed EQ-5D values above 0.9. the respiratory symptoms domain was not a significant predictor of EQ-5D utility in any of the models. Given the focus of respiratory symptoms in CF trials it may be worth exploring the potential to increase sensitivity in utility scores by developing a condition-specific preference-based measure.
TA398 (27/07/2016) Lumacaftor–ivacaftor/cystic fibrosis	Angelis (2015) [49]		Cross-sectional retrospective analysis to determine the societal economic burden and HRQoL of CF patients in the UK	74 patients with CF (UK)	EQ-5D-5L collected from patients or caregivers (UK preference weights)	Patients: mean EQ-5D index score: 0.64; EQ-VAS: 62.23. Caregivers: 0.836 and 80.85	The EQ-5D-5L can be considered a cross-sectional valid generic health outcome measure reflecting the progression of CF
TA276 (27/3/2013) Colistimethate sodium and tobramycin dry powders for inhalation/Pseudomonas lung infection in cystic fibrosis	Anyanwu (2001) [50]		Cross-sectional study to examine the applicability of EQ-5D to the assessment of HRQoL in lung transplantation	87 patients awaiting lung transplantation and 255 transplant recipients (UK)	EQ-5D-3L (UK preference weights)	Waiting list: mean (SD) VAS: 35 (19); utility score: 0.31 (0.31). Post-transplantation: mean (SD): 0–6 months: VAS: between 67 (17) and 79 (10); utility score: between 0.67 (0.15) and 0.69 (0.31); 7–18 months: VAS: between 65 (17) and 79 (17); utility score: between 0.66 (0.21) and 0.85 (0.17); 19–36 months. VAS: between 65 (20) and 79 (13); utility score: between 0.65 (0.24) and 0.86 (0.12); > 36 months: VAS: between 60 (19) and 79 (19); utility score: between 0.61 (0.31) and 0.87 (0.20)	This study has shown that QoL in all five EQ-5D domains is better in the transplanted group than in those on the waiting list, which suggests that the EQ-5D is responsive to changes in QoL resulting from symptomatic improvement after lung transplantation
	Bell (2019) [55]		Cross-sectional study to investigate the impact of ivacaftor (IVA) and standard of care (SOC) on HRQoL in groups of CF patients with G551D and F508del mutations	209 patients with CF aged ≥ 12 years, or aged 6–11 years with caregiver support (France, UK, Germany, Australia, and Ireland)	EQ-5D-5L	G551D/IVA group (*n* = 72): mean (SE) EQ-5D index score and VAS: 0.90 (0.02) and 75.7 (1.8). F508del/SOC group (*n* = 137): 0.81 (0.02) and 70.0 (1.4)	The simplicity and low costs associated with the EQ-5D-5L confer potential for more widespread use and capture of longitudinal data
	Bleisch (2019) [57]		3-year cohort study to assess HRQoL trajectories after lung transplantation	27 lung transplants recipients, of which 11 with CF and 4 with IPF (Switzerland)	EQ-5D-3L	Mean (± SD): pre-transplant: 70.43 (± 17.70); two weeks post-transplant: 62.17 (± 25.75); three months post-transplant: 85.22 (± 15.04); six months post-transplant: 87.39 (± 13.22); three years post-transplant: 89.57 (± 12.24)	EQ-5D is a generic, preference-based measure. A disease-specific measure could be more sensitive to health changes than a generic one. The EQ-5D was chosen as the outcome measure due to its being easy to complete and its previous use in lung transplant recipients.
	Bradley (2013) [59]		Longitudinal study to discover the health status and healthcare utilization associated with PEs in CF	94 patients (aged ≥ 16) with CF and chronic *P. aeruginosa* (UK)	EQ-5D-3L (UK preference weights)	EQ-5D utility index means were 0.85, 0.79 and 0.60 for no, mild and severe PEs, respectively	The EQ-5D correlated strongly with disease-specific domains of the CFQ-R. The EQ-5D allows for comparisons with other respiratory populations and QALY calculation for cost-effectiveness analyses of inhaled antibiotics treatments in CF.
	Busschbach (1994) [62]		Retrospective cohort study to measure the QoL before and after bilateral lung transplantation in patients with CF	6 patients with CF before and after lung transplantation (Netherlands)	Standard gamble, time trade-off, EQ-VAS	In general, the utility of the health states decreases when one has entered the transplant window. After transplantation, the utility increases to a level above the health state before the transplant window	The standard gamble seems to produce the highest values for the health states. The EQ-VAS produces relatively low values.
	Chevreul (2015) [67]		Retrospective cross-sectional study to estimate the economic burden and HRQoL associated with CF in France	166 patients (75 adults and 91 children aged above 5) with CF and 40 carers (France)	EQ-5D-5L collected from patients (or their legal representative in 81 children) and carers (mapping from the French EQ-5D-3L value set)	The average utility for a patient with CF was 0.730 and lower in adults (0.667 vs. 0.783 in children, *p* = 0.0015). The average utility for carers was 0.761 with no difference if they looked after an adult or a child (utility of 0.742 and 0.765 respectively, *p* = 0.7904). The average VAS was 71.9 for patients and 78.95 for carers	The advantage of the EQ-5D is that it is a generic tool which allows researchers to elicit country-specific utilities which can then be used in cost-effectiveness analysis and decision-making, but the questionnaire was developed for an adult population and is meant to be self-reporting. However, other studies have used the EQ-5D in children and parent-proxy ratings have also been shown to be both feasible and valid.
	Chevreul (2016) [66]		Cross-sectional study to estimate the economic burden and HRQoL of patients with CF and their caregivers in Europe	649 patients (357 adults and 292 children) and 271 caregivers from eight European countries	EQ-5D collected from patients and caregivers (country-specific value set when available)	In adults, mean utility fell between 0.640 and 0.870, and VAS between 46.0 and 69.7. Mean utility in caregivers was between 0.663 and 0.919 (VAS: 61.5–84.9)	The use of the EQ-5D has the advantage that it is a generic tool which allows researchers to elicit country-specific utilities which can then be used in cost-effectiveness analysis and decision-making
	Czyzewski (1994) [69]		Baseline data of an observational study assessing the impact of an educational intervention for patients with CF and their families to determine the validity of QWB scale	199 patients (younger than 18 years) and their primary caregiver (US)	QWB	QWB (total score): caregiver report (*n* = 199): mean (± SD): 0.79 (± 0.09); adolescent report (*n* = 55): 0.76 (± 0.08)	The low to moderate correlations between QWB scores from parent and adolescent reports suggest that respondents are not interchangeable. In general, overall correlations between the QWB and other indicators of physical and psychosocial functioning were low. These call into question the use of QWB as outcome measure in the paediatric CF population.
	Dewitt (2012) [72]		Analysis of data collected alongside a 48-week multicentre clinical trial evaluating the safety and efficacy of denufosol vs placebo to characterize resource use, direct medical costs, indirect costs, and utilities	352 participants (5 years or more), US and Canada	HUI2/3 completed by participants (if aged above 14) or by parents /caregivers as proxies for participants aged below 14; VAS (0–100)	HUI2/3 utility score [mean (SD)]: 0.90 (0.14); VAS: 88.3 (12.2). Mean (95% CI) change at 48 weeks: HUI2/3: 0.01 (–0.013; 0.031); VAS: 1.4 (–0.47; 3.33)	None
	Dunlevy (1994) [74]		Longitudinal study (8 weeks) to assess the effect of low impact aerobic exercise on patients work capacity, oxygen uptake and QoL	6 patients (US)	QWB	Mean (± SD) QWB: pre-exercise: 0.74 ± 0.09; post-exercise: 0.74 ± 0.02 (*p* = 1)	The lack of improvement in QWB may be related to the relative weight of the symptom/problem subscale, since only the perceived worst symptom/problem contributes to the rating
	Eidt-Koch (2009) [75]		Cross-sectional multi-centre study to evaluate the validity of the EQ-5D-Y as a generic health outcome instrument in children and adolescents with CF	96 patients (between 8 and 17 years) in Germany	EQ-5D-Y	Several low to strong correlations between the dimensions of the EQ-5D-Y and the scales of the CFQ for children, their parents and adolescents could be found. The mean VAS for 8–13-year-old patients was 85.4 (SD 16.4), the mean VAS for 14–17-year-old patients was 79.4 (SD 13.2)	The EQ-5D-Y can be considered a cross-sectional valid generic health outcome instrument which reflects differences in health according to the progression of the life-long chronic disease CF
	Fitzgerald (2005) [78]		Crossover, randomized, and placebo-controlled trial of dornase alpha before and after physiotherapy to assess the change in predicted % of FEV1, a composite QWB score, and the maximum oxygen consumption, determined using a shuttle testing	52 patients (5–18 years) in Australia	QWB	A significant period effect was demonstrated in QWB (*p* = 0.03), suggesting that on average a higher quality of well-being was achieved in period 1 (visit 2: least squares mean = 0.778; standard error = 0.008), compared with period 2 (visit 4: least squares mean = 0.752; standard error = 0.008)	None
	Fitzgerald (2018) [77]		Cross-sectional study to examine the caregiver burden in mothers and fathers of young children with CF	189 mothers and 137 fathers of young children with CF (Ireland)	CarerQoL-7D + VAS (0–10). The utility score (US) is a weighted average of the subjective burden derived from the CarerQol-7D (0–100) using utility wights from the UK population (higher US indicates less burden).	Fathers had a significantly higher median utility and VAS scores than mothers [utility: 89.2 (IQR: 79.6–96.5) vs. 84.7 (IQR: 74.5–88.0) *p* < 0.001; VAS: 8.0 (IQR: 7.0–9.0) vs 7.0 (IQR: 6.0–9.0)]	This study found that the CarerQol was a concise questionnaire easily completed by parents and appears to have some external validity
	Gold (2019) [81]		Use of a pilot observational trial/prospective observational cohort study (STOP study) data to evaluate associations between 8 symptom-based questions from the CFRSD–CRISS and the EQ-5D-5L summary score	169 patients with PEs in CF from 11 US centres	EQ-5D-5L (US population weights)	The study did not find significant correlations between the CFRSD-CRISS and EQ-5D-5L summary scores	The EQ-5D-5L alone is not a meaningful way to assess quality-of-life in future cost-effectiveness studies among CF patients with PEs. Other preference-weighted measures besides the EQ-5D-5L could be explored, such as the SF-6D, the HUI3, or perhaps a CF-specific instrument that is preference-weighted.
	Groen (2004) [82]		Cost–utility study of lung transplantation	(number of patients not specified) Netherlands	EQ-5D	On the waiting list, average utility values were 0.55 during the first 6 months, 0.50 between 6 and 9 months, 0.45 between 9 and 12 months, and 0.40 after 1 year. After transplantation, average utility values ranged from 0.69 1 month after transplantation to between 0.83 and 0.85 from 3 to 12 months after transplantation. During the next year, utility rose to 0.91. In any phase, the utility during the final 3 months before a patient died was 0.31	None
	Iskrov (2015) [90]		Cross-sectional study to determine the economic burden and HRQoL of patients with CF	23 patients and 17 caregivers (Bulgaria)	EQ-5D-3L (UK value set)	Median health utility: patients: 0.592 (IQR: –0.385 − 0.768); carers: 0.725 (IQR: 0.516 – 0.822). Median VAS: patients: 50 (IQR: 10 – 80); carers: 70 (IQR: 40 – 83)	None
	Janse (2005) [92]		Cross-sectional study to investigate the differences in perception of HRQoL between parents of chronically ill children and paediatricians	37 paediatricians and 279 parents of children with chronic conditions (including CF) in the Netherlands	HUI3	The agreement between parents and paediatricians for the overall utility score was 9%. Parents: 0.80 (median); –0.15 to 1.00 (min–max). Paediatricians: 0.93 (median); –0.13 to 1.00 (min–max)	None
	Janse (2008) [91]		Cross-sectional study to investigate the differences in perception of HRQoL among children, parents, and paediatricians	19 paediatricians and 60 chronically ill patients (aged 10–17), including 22 CF patients, and their parents (Netherlands)	HUI3	Overall utility score: paediatrician: 0.88; parent: 0.76; child: 0.69	We have chosen the HUI3 because we expected the attributes of the HUI3 to match well with major complaints of these patients. Hearing could be affected by the use of antibiotics and therefore could be relevant in patients with CF.
	Johnson (2000) [93]		Prospective observational study to examine the HRQoL of adults with CF using available generic instruments	59 adult patients (Canada)	EQ-5D-3L (UK value set)	EQ-VAS scores (74.4) were generally lower than published normal scores for this instrument (Alberta general population norms: 81.8)	None
	Munzenberger (1999) [116]		Longitudinal study to determine the responsiveness and validity of the QWB scale in children and adolescents with CF	20 children and adolescents with CF (US)	QWB	Mean (SD) QWB: before treatment: 0.611 (0.075); after treatment: 0.701 (0.07); 6 months: 0.672 (0.085); 12 months: 0.672 (0.066)	We found a significant increase in QWB scores from before to after treatment of an acute exacerbation of pulmonary disease, suggesting that the scale is a responsive measure of quality-of-life outcome of such an event. However, the QWB scale is a generic QoL instrument and may not be sensitive enough to detect changes in function domains in patients with CF. In addition, the number of patients involved in this trial was relatively small. A much larger sample may be required to detect differences in function scores when using this instrument. We conclude that the QWB scale shows both responsiveness and validity during an acute exacerbation of pulmonary disease in patients with CF. This study is limited primarily by the small number of patients. We suggest additional studies to investigate responsiveness, reproducibility, and longitudinal validity of the QWB scale.
	Orenstein (1989) [122]		Cross-sectional study to establish the construct validity of the QWB scale in CF patients	44 patients (7–36 years) in US	QWB completed by patients (if aged above 14) or by patients and parents togethers (if aged between 10 and 14) or by parents (if aged below 10)	None (only correlations coefficients)	The QWB scale is an objective measure that is significantly correlated with measures of performance and pulmonary function and has substantial validity as an outcome measure in CF patients
	Orenstein (1990) [121]		Longitudinal study (before and after 2-week treatment) to see if the QWB scale could capture change over time	28 patients (aged > 10 years) in US	QWB	QWB scores generally improved, with a mean change of 0.104 ± 0.122	The QWB can track changes in general well-being in CF patients over a brief time and detect changes associated with PE and its treatment and allows for comparison with other conditions
	Orenstein (1991) [120]		Case report study to evaluate change in overall wellbeing over time	2 patients (a 3-year-old girl and a young man) with CF (US)	QWB	QWB scores (child): prior to transplantation: 0.543; after transplantation: 0.899. QWB scores (young man): prior to transplantation: 0.6; after transplantation: 0.9)	None
	Petrou (2009) [125]		Use of data from the Disability Survey 2000 to augment previous catalogues of preference-based HRQoL weights by estimating preference-based HUI3 multi-attribute utility scores associated with a wide range of childhood conditions	2236 children with chronic conditions, including 30 with CF (England and Scotland)	HUI3 (caregiver proxy-reported)	Mean HUI3 utility score: 0.384 (± 0.336). In CF subgroup the mean score is 0.728. The mean HUI3 utility score for children of the same age is 0.929 (± 0.129)	The principal caregiver was considered the appropriate subject for the task as pilot research had indicated that the comprehension level for the HUI was somewhat high for our paediatric sample where a number of children have developmental disabilities
	Ramsey (1995) [129]		Cost-effectiveness analysis of lung transplantation	52 patients (24 on the waiting list and 28 posttransplant), of which 7 with CF (US)	Standard gamble	Mean (± SD) utility: waiting list: 0.65 ± 0.26; posttransplant (≤ 4 months): 0.73 ± 0.24; posttransplant (> 4 months): 0.89 ± 0.15	None
	Santana (2012) [133]		Cross-sectional data (at 2 years of lung transplantation) of a prospective study to assess whether HRQoL differs among diagnosis groups	214 lung transplant recipients (of which 39 with CF) at 2 years of lung transplantation (Canada)	HUI3	CF group: mean (SD) HUI overall score (at 2 years post transplantation): 0.74 (0.19)	None
	Selvadurai (2002) [134]		Randomized study to compare groups performing aerobic and resistance training with a control group in terms of fat-free mass, pulmonary function (FEV1, FVC), lower limb strength and QoL	66 children with CF (8–16 years) admitted to hospital with an infectious PE (Australia)	QWB	Baseline QWB score [mean (SD)]: aerobic training: 0.62 (0.28); resistance training: 0.60 (0.26); controls: 0.62 (0.29). Aerobic training produced significant improvements in QWB (+ 14.28%)	None
	Singer (2015) [137]		Prospective cohort study to investigate HRQoL in lung transplant recipients	387 transplanted patients (of which 83 with CF) in Canada	EQ-5D, standard gamble, VAS	Transplantation conferred large improvements in all HRQL measures [units (95% CI)]: EQ-5D of 0.27 (0.24–0.30), SG of 0.48 (0.44–0.51), and VAS of 44 (42–47)	None
	Singer (2017) [136]		Prospective cohort study to study the effect of lung transplantation on HRQoL	211 transplanted patients of which 19 with CF (US)	EQ-5D-3L	CF group (baseline): EQ-5D: 0.60 (0.44–0.78); EQ-VAS: 29 (20–30); CF group (change after lung transplantation): EQ-5D: 0.30 (0.22–0.39); EQ-VAS: 43.0 (36.8–49.3)	None
	Solem (2016) [139]		Use of data from a 48-week randomized, placebo-controlled study of ivacaftor to examine the impact of PEs and lung function on generic measures of HRQoL	161 patients ≥ 12 years with CF and a G551D-CFTR mutation (US)	EQ-5D-3L (UK value set)	EQ-5D index (mean, [SE]): no lung dysfunction: 0.931 (0.023); mild: 0.923 (0.021); moderate: 0.904 (0.018); severe: 0.870 (0.020). VAS score: no lung dysfunction: 85.2 (2.0); mild: 82.3 (1.8); moderate: 76.8 (1.6); severe: 73.3 (1.8)	In our analyses, ceiling effects were high, particularly for the EQ-5D index and in patients with no lung dysfunction or mild lung dysfunction as well as among those with less severe disease (i.e., patients who did not experience PEs). It should be noted that the EQ-5D index at the time of study initiation was high (mean ≈ 0.93) leaving little room for improvement with study treatments. While both EQ-5D and VAS are generic measures, they provide complementary information; the use of the EQ-5D index alone (a generic HQRL measure) may limit characterization of disease burden and health gains in patients with CF. The EQ-VAS measure showed greater ability to discriminate disease severity than the EQ-5D index. Some dimensions of the EQ-5D, particularly self-care, are less likely to be impacted by CF. Moreover, the EQ-5D was designed for use in populations 18 years of age or older, whereas this study included adolescent patients as young as 12 years of age.
	Suri (2001) [141]		Open cross-over 12-week trial to compare hypertonic saline and alternate-day or daily recombinant human deoxyribonuclease in terms of FEV1, FVC, number of PEs, weight gain, QoL, exercise tolerance and costs	47 children with CF aged between 5 and 18 (UK)	QWB	Mean QWB at baseline (SD; range): 0.61 (0.12; 0.35–0.84)	None
	Vasiliadis (2005) [148]		Cost–utility analysis of lung transplantation (including a cross-sectional study to estimate utilities)	105 patients (34 candidates and 71 recipients) in Canada	Standard gamble	Mean (± SD) difference between post-transplant recipient reported utility and candidate reported utility (CF and bronchiectasis subgroup): waiting list: 0.11 ± 0.09 (*n* = 8); after transplantation, first year: 0.72 ± 0.11 (*n* = 10); second year: 0.82 ± 0.12 (*n* = 8); third year: 0.84 ± 0.19 (*n* = 2); fourth year: 0.87 ± 0.16 (*n* = 3); > fifth year: 0.61 ± 0.12 (*n* = 7)	The SG assumes that subjects are neutral toward probability risks. In this study, candidates may have been more ready to accept a risk, thus underestimating the utility associated while waiting.
	Whiting (2014) [150]		Cost-effectiveness analysis of ivacaftor for the treatment of CF	Patients aged ≥ 6 years who have at least one G551D mutation in the CFTR gene (UK)	EQ-5D (US and European value sets)	Utility values by percentage predicted FEV1: normal (FEV1 ≥ 90%): 0.97; mild (FEV1 70–89%): 0.95; moderate (FEV1 40–69%): 0.93; severe (FEV1: < 40%): 0.91	None
	Yi (2003) [153]		Cross-sectional study to assess health values in adolescents with CF	65 adolescents (12–18 years) with CF (US)	HUI2, VAS, standard gamble, and time trade-off (VAS results were normalized to a 0.0–1.0 scale)	The mean (± SD) TTO utility was 0.96 (± 0.07) and the mean (± SD) SG utility was 0.92 (± 0.15). The mean (± SD) global utility estimate derived from the HUI2 was 0.83 (± 0.16). The mean (± SD) VAS was 0.76 (± 0.2).	The HUI2 did not appear to discriminate well between levels of health. The mean HUI2 global utility estimate was significantly lower than both the TTO and SG scores and did not correlate with either score. Nevertheless, because the issue of fertility is important in CF, an advantage of the HUI2 is that is addresses fertility. The utilities derived by the TTO and SG methods correlated well with each other and mean values were similar.
HST2 (16/12/2015) Elosulfase alfa/Mucopolysaccharidosis type IVa	Hendriksz (2014) [86]		Cross-sectional survey to assess the global burden of disease among patients with Morquio A, including the impact on mobility/wheelchair use, HRQoL, pain and fatigue and the interaction between these factors	27 adults (≥ 18) and 36 children (7–17 years) from Brazil, Colombia, Germany, Spain, Turkey, and UK	EQ-5D-5L	The HRQoL utility values were 0.846, 0.582 and 0.057 respectively in adults not using a wheelchair, using a wheelchair only when needed and always using a wheelchair; values were 0.534, 0.664 and –0.180 respectively in children. In both adult patients and children, the HRQoL was most negatively affected in the domains mobility, self-care, and usual activities.	In the adult patient group, the EQ-5D pain/discomfort domain scores did not follow the pain severity scores obtained by the BPI-SF for the different mobility categories. Most likely, this is due to the fact that the pain/discomfort score of the EQ-5D-5L is based on a single question whereas the BPI-SF comprises different questions to assess pain severity and will be able to capture more subtle differences between patients.
HST2 (16/12/2015) Elosulfase alfa/Mucopolysaccharidosis type IVa	Hisashige (2011) [87]		Cross-sectional survey to estimate preference-based HRQoL (i.e., utility) in patients with MPS using proxy respondents	93 medical students and 6 medical experts (Japan)	Direct measurement (i.e., VAS, standard gamble, and time trade-off)	MPS IV (Morquio syndrome), mean (SD): medical students: VAS: 0.22 (0.22); TTO: 0.47 (0.22); SG: 0.54 (0.27); medical experts: VAS: 0.39 (0.21); TTO: 0.52 (0.28); SG: 0.62 (0.28);	In this study, the patients of MPSs were not used as the respondents, since they are often children with mental retardation, who would have difficulty in evaluating utility of their own health states. Health professionals are used as surrogate respondents for the patients. Utility values among medical experts were quite similar to those among medical students. However, the sample size of medical experts was very small. Any surveys using parent proxies and/or a sample of the general population are encouraged. Indirect approaches (e.g., EQ-5D) would be more practical with larger samples but their validity needs to be examined in comparison with direct measurement results.
	Péntek (2016) [124]		Multi-country, cross-sectional survey to assess the HRQoL of patients with MPS and their caregivers	120 patients and 66 caregivers (seven European countries)	EQ-5D-3L collected from patients and caregivers (country-specific societal value sets)	Patients’ average (SD) EQ-5D and VAS scores varied across countries from 0.13 (0.43) and 0.43 (0.30), and from 30.0 (28.3) and 62.2 (18.3), respectively Caregivers’ average (SD) EQ-5D and VAS scores varied from 0.39 (NA) and 0.94 (0.11), and from 45.0 (NA) and 84.4 (12.1), respectively	None
HST2 (16/12/2015) Elosulfase alfa/Mucopolysaccharidosis type IVa	Pintos-Morell (2018) [126]		Prospective real-world study to evaluate the effectiveness of ERT with elosulfase alpha in terms of 6-MWT, 3-MSCT, urinary glycosaminoglycans and HRQoL	7 patients with MPS-IVA (Spain)	EQ-5D-5L	EQ-VAS (range): baseline: 25–60; 8 months: 45–75	We use the EQ-5D-5L questionnaire, as recommended by the international guidelines for the management and treatment of MPS IVA
HST8 (10/10/2018) Burosumab/X-linked hypophosphatemia	Forestier-Zhang (2016) [80]		Use of cross-sectional data from a prospective cohort study to describe HRQoL and conduct a cost-utility analysis of a hypothetical treatment for a rare bone disease	24 patients (UK)	EQ-5D-5L (English value set)	The mean health utility score was 0.648 (± 0.290) and mean VAS was 60.8 (± 26.9)	The EQ-5D-5 L is a generic HRQoL measure and may not capture specific problems for this patient group; also, the utility score is derived from the preferences of the general population and the preferences for patients with rare bone diseases may differ
HST7 (7/2/2018) Strimvelis/Adenosine deaminase deficiency- severe combined immunodeficiency	Van der Ploeg (2019) [146]		Cost-effectiveness analysis of new-born screening for severe combined immunodeficiency	None	Author’s estimates based on published literature and in consultation with clinical experts	Utility: good: 0.95; moderate: 0.75; poor: 0.5	None
TA588 (24/7/2019) Nusinersen/Spinal muscular atrophy	Belter (2020) [56]		Cross-sectional survey to collect baseline QoL results among individuals affected by SMA	478 individuals and/or caregivers of individuals affected by SMA (worldwide)	HUI3 completed by parents of affected children aged 5 and up and by affected adults aged 18 and up (children did not complete the HUI)	Overall, the average HUI3 scores ranged from –0.05 to 0.64	The HUI3 measures someone’s ability to perform daily activities, such as dressing, which is a common activity that patients with SMA would like to improve or maintain. However, not all the attributes assessed by the HUI3 may be relevant for any of the SMA population. For example, the HUI3 assesses QoL through vision and hearing attributes, which are not relevant to the manifestation of SMA across types, and the high attribute scores as shown in this analysis, demonstrate this.
	Chambers (2020) [64]		Cross-sectional cohort study to quantify the economic and HRQoL burden incurred by households with a child affected by SMA	40 children (and their parents) with SMA I, II and III (Australia)	EQ-5D-Y completed by patients or caregivers on behalf of their child if aged below 8 (EQ-5D-3L Australian valuation weights used as proxy). CarerQoL-7D (Australian valuation weights) completed by caregivers (for themselves)	Average caregiver and patient utility scores were 0.708 and 0.115, respectively. Average caregiver and patient VAS scores were 6.14 (0–10) and 66 (0–100)	None
TA588 (24/7/2019) Nusinersen/Spinal muscular atrophy	Lloyd (2019) [106]		Cross-sectional study to estimate QoL weights or utilities of different SMA states	5 clinical experts (UK)	(1) Development of SMA case histories describing Type I (infantile onset) and Type II (later onset) patients and different outcomes from treatment. (2) Valuation of case studies using EQ-5D-Y proxy reported (EQ-5D-3L UK weights used as proxy)	The SMA Type I utilities ranged from −0.33 (requires ventilation) to 0.71 (Type I patient reclassified as Type III following treatment). The SMA Type II utilities ranged from − 0.13 (worsened) to 0.72 (stands/walks unaided)	The study presents some limitations. First, the accuracy of the HRQoL results was dependent on the validity or accuracy of the case study descriptions, and the ability of the experts to provide an accurate proxy assessment of HRQoL. Second, the manifestation of SMA is heterogeneous but the descriptions of SMA states are by necessity a simplification and do not reflect this variability. Third, it was only possible to recruit a small group of expert physicians. However, while the group was small, their ratings were quite consistent; physicians are also able to draw on their experience of treating numerous patients with SMA, as opposed to a parent’s experience of caring for just their child; therefore, using physicians for proxy reporting allow to avoid some bias that parental judgment of HRQoL may introduce. Finally, the assessment of HRQoL used the EQ-5D-Y, which has not been proven as valid in children as young as some patients with SMA (e.g., infantile-onset patient population). However, no other alternatives currently exist for the assessment of HRQoL in such young children or infants. Despite these limitations, the study has produced data with face validity.
TA588 (24/7/2019) Nusinersen/Spinal muscular atrophy	López-Bastida (2017) [110]		Cross-sectional and retrospective study to determine the economic burden and HRQoL of patients with SMA and their caregivers in Spain	81 caregivers of patients (1–17 years) with SMA (Spain)	EQ-5D completed by caregivers (3L proxy version for patients, 5L for caregivers)	Mean EQ-5D score: 0.16 (patients) and 0.49 (caregivers). Mean EQ-VAS: 54 (patients) and 69 (caregivers)	None
	Zuluaga-Sanchez (2019) [156]		Cost effectiveness of nusinersen for the treatment of patients with infantile-onset (ENDEAR phase III trial) and later-onset SMA (CHERISH phase III trial)	Patients and caregivers (Sweden)	The ENDEAR and CHERISH trials did not collect utility values from patients or caregivers. Patient’s utilities were derived from Lloyd. To estimate the caregiver utilities, a fixed caregiver utility value was anchored to the stabilisation of the baseline function health state (infantile-onset: 0.850; later onset: 0.815; assumption). Then, the difference in magnitude between the patient utility of the stabilisation of the baseline function health state (−0.120 in the infantile-onset and 0.04 in the later-onset) and the other health states (e.g., the utility assigned to patients in the worsened health state in the infantile-onset model was −0.240; the difference to the stabilisation of baseline function health states was 0.12) was applied to derive the caregiver utilities for each health state. The disutility was the difference between the caregivers utility and the general population utility.	Health-state caregiver disutility values: worsened: –0.160; stabilization of baseline function: – 0.040; mild improvement: –0.090; loss of later-onset SMA advanced motor function: –0.160	As the CHERISH study collected the PedsQL and a mapping algorithm for PedsQL to EQ-5D was available (Khan 2014), EQ-5D utility values were estimated for the later-onset model health states with the limitation that the patients in the CHERISH trial were substantially younger than the patients used to develop the algorithm. However, these utility values were not explored in the scenario analysis, as they lacked face validity (the utility estimated for the worst health state was 0.73, while the valuation elicited from clinicians in the study by Lloyd et al. for the same health state was − 0.13).
TA431 (25/1/2017) Mepolizumab/Severe refractory eosinophilic asthma	Lloyd (2007) [108]		Prospective study to report the impact of exacerbations on HRQoL and health utility in patients with moderate to severe asthma	112 patients (UK)	EQ-5D; ASUI	Mean (SD): ASUI: no exacerbation: 0.75 (0.20); exacerbation: 0.48 (0.27); hospitalized: 0.31 (0.22); EQ-5D utility: no exacerbation: 0.89 (0.15); exacerbation: 0.57 (0.36); hospitalized: 0.33 (0.39); EQ-VAS: no exacerbation: 76.1 (15.51); exacerbation: 56.43 (21.58); hospitalized: 49.0 (19.49)	There was some evidence of floor effect on ASUI in its ability to capture the impact of exacerbations
HST5 (28/6/2017) Eliglustat/Gaucher disease (type 1)	Clarke (1997) [68]		Cross-sectional survey to elicit utilities for three hypothetical health states of GD that are indications for treatment with alglucerase and to study the effect of the respondent’s experience with the health state of interest	Three adult populations: (1) 39 normal volunteers who denied having any significant chronic illness (US and Canada); (2) 38 chronically ill respondents (US and Canada), and (3) 32 GD patients (US)	Health state descriptions (vignettes) valued by three direct methods (i.e., TTO; SG; risk–risk trade off, RRTO). Health state descriptions were developed from medical textbooks and a videotape prepared by the NGF. Three nationally recognized physicians, six nurses who treat large numbers of patients and a number of patients verified the accuracy of the descriptions.	GD type 1 [mean ± (95% CI)]: healthy subjects: TTO: 0.87 (0.83–0.91); SG: 0.94 (0.91–0.97); RRTO: 0.32 (0.23–0.41). Chronic illness: TTO: 0.88 (0.83–0.93); SG: 0.92 (0.86–0.98); RRTO: 0.30 (0.20–0.40). GD: TTO: 0.87 (0.82–0.92); SG: 0.94 (0.91–0.97); RRTO: 0.55 (0.42–0.68)	The RRTO produced significantly lower utilities than the other two preference assessment methods and had the poorest test–retest reliability
HST5 (28/6/2017) Eliglustat/Gaucher disease (type 1)	Deegan (2011) [71]		Observational cross-sectional study to quantify the burden of residual skeletal disease after ERT and to explore putative relationships between clinical, radiologic, and biochemical factors, bone sequelae and QoL	100 adult patients with GD (UK)	EQ-5D-3L (UK preference weights)	The median health state score derived from the EQ-5D was 0.727 (CI: 0.691–0.796)	None
	van Dussen (2014) [147]		Cost-effectiveness analysis of ERT for type 1 Gaucher disease	(number of patients not specified) Netherlands	EQ-5D (UK and Dutch value sets)	Mean EQ-5D UK (95% CI): asymptomatic: 0.93 (0.89–0.97); symptoms/recovery: 0.8716 (0.8177–0.9225); splenectomy: 0.7532 (0.6768–0.8215); bone complication: 0.8614 (0.7530–0.9685); multiple complications: 0.7323 (0.6601–0.8202). Mean EQ-5D NL (95% CI): asymptomatic: 0.93 (0.89–0.97); symptoms/recovery: 0.8897 (0.8410–0.9349); splenectomy: 0.7781 (0.6990–0.8626); bone complication: 0.8882 (0.8027–0.9707); multiple complications: 0.7981 (0.7430–0.8638)	None
HST5 (28/6/2017) Eliglustat/Gaucher disease (type 1)	Flowers (1997) [79]		Cross-sectional survey to compare the use of willingness-to-pay (WTP) and standard gamble methods to elicit preferences for GD	52 healthy volunteers (US)	SG: the method interviewed subjects to determine the maximum risk of death that they would accept to avoid life with GD. WTP: was assessed as the greatest amount that a subject would pay per month to purchase an insurance policy to cover the cost of the treatment for GD.	WTP: on average, subjects were willing to pay $107 (SD: ± $99; median: $90) per month for insurance to cover the cost of treatment for GD. SG: mean utility 0.860 (SD: ± 0.192; median: 0.950)	On the whole, this implementation of the WTP method produced results on par with an existing computer-based SG method that has been shown in previous studies to be well-accepted in other clinical contexts. The WTP method may be particularly well-suited to measure preferences for conditions where it is unrealistic to trade-off life expectancy or to take a risk of immediate death (e.g., chronic conditions near normal health).
	Hadi (2018) [84]		Valuation study to estimate the utility values associated with each health state related to treatment mode of administration in GD1	100 members of the general population (UK)	Time trade-off and VAS using vignettes informed by review of relevant literature and expert clinical input	Mean (SD) TTO-derived utility and VAS values: controlled disease: 0.89 (0.11) and 71.4 (17.9); oral treatment: 0.85 (0.15) and 71.0 (17.1); intravenous treatment: 0.73 (0.20) and 50.0 (17.8). The mean EQ-5D single index score for the UK population is 0.86.	Another limitation of the study that one could raise is the absence of direct input from patients with GD in the development of the health states descriptions. However, the purpose of the study was to focus on specific known attributes related to the modes of treatment administration rather than the disease itself.
HST5 (28/6/2017) Eliglustat/Gaucher disease (type 1)	Lenert (1998) [104]		Randomized controlled trial of two search procedures (titration and “ping-pong”) using two otherwise identical computer programs that describe health states related to GD to examine the effects of changing the search procedure on utility values	62 healthy volunteers (US)	Time trade-off, standard gamble, and VAS	For both the TT0 and the SG rating tasks, the titration group had higher mean ratings (of about 0.10 and 0.15, respectively) than did the ping-pong group	The procedure used to search for subjects’ utility values strongly influences the results of preference-assessment experiments. We used GD as a model health condition because we anticipated that few subjects would have prior knowledge of the disease; hence ratings would be based on our descriptions of the health state rather than subjects’ prior experiences and/or prejudices.
	Wyatt (2012) [152]		Cost-effectiveness analysis of ERT for lysosomal storage disorders (LSDs)	175 patients (UK)	EQ-5D and SF-36 derived (SF-6D) (UK weights)	None	None
TA467 (16/8/2017) Holoclar/Limbal stem cell deficiency after eye burns	Smith (2020) [138]		Cross-sectional survey to generate utility health states for adult patients with moderate-to-severe limbal stem cell deficiency (LSCD), unilateral or bilateral, due to physical or chemical ocular burns	520 members of the general population (UK)	EQ-5D-3L with vision bolt-on scores (converted using three different UK value sets) + standard gamble	EQ-5D-3L: the mean disutility for LSCD with poor visual acuity, pain and disfigurement in both eyes compared to one eye was −0.084 (range = −0.156 to −0.045 across the value sets). The mean disutility of bilateral LSCD with pain, disfigurement, and poor visual acuity compared to unilateral LSCD with only poor visual acuity in one eye was −0.104 (range = −0.151 to −0.078). Similarly, where one eye was affected, pain and disfigurement in combination were associated with a greater mean disutility than improvements in visual acuity alone: −0.011 (range = −0.04 to 0.005). SG: mean utilities were within a narrow range (0.682–0.765). Where one eye was affected, the main driver was disfigurement: mean utility was 0.731 (0.709–0.753) compared to 0.682 (0.659–0.704) when disfigurement was removed compared to vision restored to normal. For bilateral LSCD, mean utilities were 0.693 (0.672–0.715) for normal vision and 0.75 (0.73–0.771) when disfigurement and pain were removed.	One caveat on the data generated by this study is that the raw data indicated that some participants failed to accurately imagine the LSCD vignettes prior to completing the EQ-5D-3L. This can be observed in the frequencies of individuals who selected “no problems “ in the vision domain of the EQ-5D-3L, in spite of being told they had severe LSCD in both eyes which caused poor vision. Other limitations with this study include the fact that there is little overlap between the domains on the EQ-5D-3L and the vignettes describing LSCD. The EQ-5D-3L does not contain a visual acuity domain, whereas other instruments such as the HUI3 do contain a vision domain. This argument is, however, partially mitigated by the inclusion of the EQ5D + V. Given the nature of LSCD, in particular the rarity of the condition, deriving utilities from patients with preference-based instruments such as the EQ-5D-3L and/or vignettes would not lead to robust results due to small sample size. Moreover, there are other vignette methods, for instance the TTO and discrete choice experiments that could have been applied to derive health utilities in this study.
TA606 (16/10/2019) Lanadelumab/Hereditary angioedema	Aygören-Pürsün (2016) [53]		Cross-sectional survey to conduct a mapping exercise between survey items and EQ-5D	111 patients (aged ≥ 12) with HAE (Spain, Germany, Denmark)	EQ-5D-3L (UK value set)	Mean EQ-5D index score: 0.44 (HAE attack) and 0.72 (between attacks)	The HAE-BOIS-Europe survey items selected for the manual cross-walking to the EQ-5D appear to be sufficiently comparable conceptually
	Aygören-Pürsün (2019) [52]		Randomized, single‐blind, crossover, phase 3 trial of C1 inhibitor (C1‐INH) prophylaxis to investigate the safety, efficacy and HRQoL outcomes	12 patients (7–11 years) from US, Germany, Israel, Mexico, and Romania	EQ-5D-Y	Mean EQ‐VAS change from baseline to week 9 of treatment (500 U C1‐INH, 10.4; 1000 U C1‐INH, 21.6) was greater than the minimal important difference, indicating a meaningful HRQoL change	None
	Lumry (2018) [111]		Post hoc analysis of data from placebo-controlled, cross-over phase trial (COMPACT study) to assess HRQoL outcomes in patients self-administering subcutaneous C1-inhibitor deficiency C1-INH)	90 patients (US, Canada, Australia, Czech Republic, Hungary, Israel, Italy, Romania, Spain, UK)	EQ-5D-3L	EQ-5D health state value (HSV) and VAS scores were high at baseline (HSV: mean: 0.89; SD: 0.194; VAS: mean: 82.23; SD: 17.132). VAS score changes between C1-INH(SC) and placebo at the last visit (week 14) indicated a treatment benefit with C1-INH (SC) (mean treatment difference, 8.53; 95% CI 4.10, 12.97). However, changes in HSV scores from baseline to the last visit were small (mean treatment difference, 0.04; 95% CI: -0.01, 0.08) and not suggestive of a treatment benefit with C1-INH versus placebo (mean HSV week 14: C1-INH: 0.92; placebo: 0.87)	None
TA606 (16/10/2019) Lanadelumab/Hereditary angioedema	Nordenfelt (2014) [117]		Retrospective registry study to investigate the burden of disease and perceived health state in Swedish patients with HAE	103 patients (Sweden)	EQ-5D-5L	Mean ± SD EQ-5D utility: attack-free state (today): 0.825 ± 0.207; last attack state: 0.512 ± 0.299	In the absence of a validated disease-specific instrument in 2011, the EQ-5D-5L was selected as a recognized tool for assessing health status, providing an opportunity to compare the impact of HAE with the impact of other conditions. Results from our survey suggest that EQ-5D-5L could be used to measure HAE disease impact.
	Nordenfelt (2017) [118]		Cross-sectional registry study to investigate HRQoL in Swedish patients with HAE by combining different instruments (i.e., EQ-5D-5L, RAND-36 and AE-Qol)	64 patients (Sweden)	EQ-5D-5L	Median (range) EQ-5D index value: 0.84 (–0.02 to 1.00); EQ-VAS score: 80 (25–100)	The AE-QoL is more disease specific in HAE than the generic instruments EQ-5D-5L and RAND-36; however, the latter highlights the pain aspect, whereas the AE-QoL does not
TA443 (26/4/2017) Obeticholic acid/Primary biliary cholangitis	Åberg (2011) [47]		Cost–utility analysis of liver transplantation	64 patients with chronic liver disease (of which: 16 PSC and 8 PBC) in Finland	15D	Median 15D scores: 0.798 (before LT) and 0.898 (after LT)	None
	Åberg (2012) [46]		Cross-sectional study to investigate HRQoL among all adult liver transplantation (LT) patients in Finland	353 LT patients (of which 56 PSC and 72 PBC) in Finland	15D	Age-adjusted mean 15D scores: 0.886 (PSC) and 0.882 (PBC). General population: 0.914	None
TA443 (26/4/2017) Obeticholic acid/Primary biliary cholangitis	Haapamaki (2015) [83]		Prospective study to assess HRQoL of patients with PSC, and to compare it with that of the general population	341 patients with PSC (Finland)	15D	The mean 15D score was 0.930 (median 0.945). No significant difference was seen in 15D scores between PSC patients and general population	None
	Longworth (2003) [109]		Cost-effectiveness analysis of liver transplantation	347 adult patients (≥16 ), of which 70 with PSC, assessed for liver transplantation in UK	EQ-5D (UK tariff)	Mean EQ-5D scores for the patient sample are lower at all timepoints compared with age/gender adjusted UK population mean scores	None
	Rice (2020) [130]		Use of data from the UK-PBC cohort to estimate costs and HRQoL	2240 patients (10% of all UK PBC patients)	EQ-5D-5L (UK cross-walk value set)	No PBC-related symptoms or complications: 0.917. With symptoms: between 0.6 and 0.8	None
	Younossi (2001) [154]		Cross-sectional study to test the validity of a widely used utility measure in CLD	120 patients with CLD (including 16 with PSC) in the US	HUI2	Mean (± SD) utility: 0.78 ± 0.22	The HUI performs well in this population (patients with mild disease have an appreciable decrement in utilities and patients with more severe disease have a major decrement). The results differ in important ways from previous estimates of utilities by experts.
TA491 (22/11/2017) Ibrutinib/Waldenstrom’s macroglobulinaemia	Dimopoulos (2017) [73]		Open-label sub study of an international, multicentre, phase 3 trial to assess the efficacy and safety of ibrutinib in a population with rituximab-refractory disease	31 patients (aged 18 and older) in seven countries	EQ-5D-5L	A clinically meaningful improvement from baseline in the EQ-VAS were reported at all post-baseline visits	None
TA379 (27/1/2016) Nintedanib/Idiopathic pulmonary fibrosis	Bloem (2020) [58]		Cross-sectional study to determine the prevalence of severe fatigue in patients with ILD (IPF or pulmonary sarcoidosis) and to evaluate the association between fatigue and clinical parameters	117 patients with ILD (59 with IPF) in the Netherlands	EQ-5D-5L	IPF: index value: 0.74 ± 0.18; VAS: 63.3 ± 16.5	The generic EQ-5D-5L is not an ILD-specific questionnaire for QoL and consequently it does not capture disease-specific effects of ILD. However, the EQ-5D-5L is validated in patients with different lung diseases and will make comparisons between patients with different lung diseases and the general population possible.
	Chang (1999) [65]		Cross-sectional study to assess the validity of several generic and respiratory-specific quality-of-life instruments in patients with interstitial lung disease	50 ILD patients, of which 33 with IPF (US)	QWB	Median (IQR) QWB score: 0.65 (0.58–0.71)	Although the study population represented the full spectrum of disease severity, their QWB scores fell within a relatively narrow range. Because of the lesser variability in QWB scores, this generic questionnaire may be less successful in distinguishing between patients with interstitial lung disease.
	Han (2013) [85]		Randomized trial to determine whether sildenafil improves 6-MWT in subjects with IPF and right ventricular dysfunction	119 patients with IPF and right ventricular dysfunction (US)	EQ-5D	Subjects treated with sildenafil experienced greater improvement in EQ-VAS scores (17.9 points; *p* = 0.04) than subjects receiving placebo	EQ-5D may be more sensitive to short-term QoL changes than the SF-36 in IPF
TA379 (27/1/2016) Nintedanib/Idiopathic pulmonary fibrosis	King (2011) [94]		Randomized (2:1) placebo-controlled trial of bosentan to measure its effects on time to IPF worsening (i.e., a confirmed decrease from baseline in FVC > 10% and diffusing capacity of the lung for carbon monoxide > 15%, or acute exacerbation of IPF) or death, HRQoL, dyspnoea, safety, and tolerability	616 patients from 19 countries	EQ-5D	EQ-5D score (baseline): bosentan (*n* = 407): 0.758 ± 0.185; placebo (*n* = 209): 0.718 ± 0.242. EQ-5D score (1 year): bosentan: 0.660 ± 0.386; placebo: 0.656 ± 0.366. VAS (baseline): bosentan (*n* = 407): 70.4 ± 18.7; placebo (*n* = 209): 69.5 ± 19.4. VAS (1 year): bosentan: 65.9 ± 24.0; placebo: 66.4 ± 23.2	None
	Kotecha (2016) [95]		Cross-sectional study to assess whether patient confidence is associated with HRQoL	75 patients with IPF (UK)	EQ-5D	Patient confidence was significantly positively associated with HRQoL	None
	Kreuter (2017) [96]		INSIGHTS-IPF registry to describe associations of various QoL instruments between each other and with patient characteristics at baseline	623 patients (Germany)	EQ-5D	Mean (SD) EQ-VAS: from 65.6 (20.0) for no comorbidity to 50.9 (18.3) for ≥ four comorbid diseases	None
	Martinez (2014) [113]		Randomized placebo-controlled trial of N-acetylcysteine (NAC) to assess the change in FVC over a 60-week period	264 patients (US)	EQ-5D	VAS (baseline): NAC (*n* = 133): 78.3 ± 15.0; placebo (*n* = 131): 77.7 ± 14.3. VAS change at 60 weeks: NAC: 0.9; placebo: –3.3	None
	Porte (2018) [127]		Cost–utility analysis to evaluate the efficiency of nintedanib in comparison with all available alternatives	(number of patients not specified) France	EQ-5D (French value set)	Mean (SD) EQ-5D utility value: between 0.30 (0.36) and 0.82 (0.35) depending on forced vital capacity (FVC)	None
TA379 (27/1/2016) Nintedanib/Idiopathic pulmonary fibrosis	Salisbury (2020) [132]		Use of data from the IPF Prospective Outcomes (IPF-PRO) Registry to investigate patterns of use of antifibrotic medications	782 patients (US)	EQ-5D	Median EQ-5D score (25th–75th percentile) Treated: 0.8 (0.7–1.0); untreated: 0.8 (0.7–1.0). VAS score: Treated: 75 (60–85); 79 (67–90)	None
	Shulgina (2013) [135]		Randomized placebo-controlled trial of co-trimoxazole to measure FVC (primary endpoint), DLCO, EQ-5D utility, 6-MWT and dyspnoea score (secondary endpoints)	181 patients (UK) with fibrotic idiopathic interstitial pneumonia (89% diagnosed as definite/probable IPF)	EQ-5D-3L (UK value set)	Co-trimoxazole treatment resulted in a significant improvement in EQ5D-based utility (mean difference: 0.12 (95% CI: 0.01 to 0.22))	None
	Sundh (2018) [140]		Prospective, multicentre, cohort study to validate the Swedish version of the Dyspnoea-12 (D-12)	35 patients with IPF (Sweden)	EQ-5D-5L (UK value set)	None	None
	Szentes (2018) [142]		Baseline HRQoL data of an ongoing observational study (HILDA) to compare the disease-specific K-BILD and the generic EQ-5D-5L in patients with ILD	229 ILD patients including 55 patients with IPF (Germany)	EQ-5D-5L (Germany-specific experience-based value set -EBVS)	Mean (SD) EQ-5D-5L score: 0.66 (0.17); mean (SD) VAS score: 61.4 (19.1)	The EQ-5D-5L has properties similar to the K-BILD and hence allowing its use in the ILD disease group. K-BILD reacted more sensitively to ILD-specific aspects of HRQL rendering it a valuable complementary measure to the generic EQ-5D-5L.
	Po Yu Tsai (2020) [143]		Sub study of a prospective multicentre observational cohort study (CARE-PF) to demonstrate feasibility of use and calculate MID of the EQ-5D-5L and its associated EQ-VAS in patients with fibrotic ILD	1816 patients with fibrotic ILD, of which 472 with IPF (≥ 18 years) in Canada	EQ-5D-5L (Canadian value set)	EQ-5D-5L score [mean (± SD)] at baseline: 0.77 ± 0.19. EQ-VAS: 68 ± 19. There were 340 patients (19%) with perfect scores of 0.949; of these, only 7% had perfect VAS scores	The findings support the use of EQ-5D-5L in patients with fibrotic ILD, but with the limitation that there is a significant ceiling effect in mild disease. Further validation in other countries is required.
TA379 (27/1/2016) Nintedanib/Idiopathic pulmonary fibrosis	Tzanakis (2005) [145]		Cross-sectional study to determine whether the HRQoL is affected in patients with IPF	25 patients (aged 41–72 years) and 30 healthy subjects (Sweden)	QWB	Mean (± SD) QWB values: patients: 0.59 (± 0.29); controls: 0.866 (± 0.05); *p* < 0.001	We did not find any correlation between QWB and physiological parameters
	Wapenaar (2017) [149]		Cross-sectional study to translate and validate the King’s Brief Interstitial Lung Disease (K-BILD)	195 patients with ILD, of which 108 with IPF (France, Italy, Netherlands, Sweden)	EQ-5D-5L	Mean (SD) EQ-5D index value (IPF only): 0.66 (0.23); VAS: 60.8 (18.9)	None
	Zisman (2010) [155]		Randomized, placebo-controlled trial to test whether sildenafil would improve 6-MWT, dyspnoea, and QoL in patients with advanced IPF	180 patients (89 in the sildenafil and 91 in the placebo group) in the US	EQ-5D	Baseline mean (± SD) EQ-5D score: sildenafil: 0.71 ± 0.24; placebo: 0.74 ± 0.19. EQ-VAS: sildenafil: 66.49 ± 17.45; placebo: 67.66 ± 16.98. Mean change (95% CI) at week 12: EQ-5D score: sildenafil: –0.01 (–0.06 to 0.03); placebo: –0.03 (–0.08 to 0.01); VAS: sildenafil: 0.48 (–3.10 to 4.06), placebo: – 1.81 (–5.34 to 1.73)	None
HST3 (20/7/2016) Ataluren/Duchenne muscular dystrophy	Bray (2017) [60]		Postal survey to evaluate the validity of proxy HRQoL measures in the context of paediatric mobility impairment	13 child–parent dyads (13 children with mobility impairments, 13 parent proxies) of which 1 child with muscular dystrophy and his/her parent (England and Wales)	EQ-5D-Y and HUI2/3	Child self-report: EQ-5D-Y: 0.24 ± 0.30; VAS: 79.54 ± 15.01; HUI2: 0.53 ± 0.07. HUI3: 0.22 ± 0.09 Parent proxy: EQ-5D-Y: 0.01 ± 0.14; VAS: 75.77 ± 14.70; HUI2: 0.49 ± 0.09. HUI3: 0.16 ± 0.10	The EQ-5D-Y did not sufficiently measure HRQoL in accordance with how the sampled children and parents defined overall health status. This is likely due to general population health state preferences being unrepresentative of how children with mobility impairments (and their parents by proxy) value their own health state. The simple descriptive system of the EQ-5D-Y lacks nuance for children with mobility impairments. For instance, the EQ-5D-Y mobility domain has no consideration for mobility beyond walking and thus automatically discounts the HRQoL of a mobility impaired child. Overall, EQ-5D and HUI lack sufficient sensitivity to be used appropriately in mobility impaired populations. Significant differences were found between child and parent proxy measures, and yet they were also correlated.
	Cavazza (2016) [63]		Cross-sectional study to determine the economic burden from a societal perspective and the HRQoL of patients with DMD in Europe	422 (268 adult patients and 154 caregivers) from eight European countries	EQ-5D collected from patients or caregivers	The average EQ-5D index score and VAS were 0.24 and 50.5 (adult patients) and 0.71 and 74.7 (caregivers)	None
HST3 (20/7/2016) Ataluren/Duchenne muscular dystrophy	Landfeldt (2014) [102]		Cross-sectional study to estimate the total cost of illness and economic burden of DMD	770 patient-caregiver pairs (Germany, Italy, UK, US)	HUI (for patients) and EQ-5D (for caregivers)	Mean proxy-assessed HUI–derived utility was estimated at 0.45 (95% CI: 0.41–0.51), 0.52 (0.45–0.58), 0.43 (0.39–0.47), and 0.45 (0.42–0.49) for patients from Germany, Italy, UK, and US, respectively. Corresponding EQ-5D utility estimates for the caregivers were 0.79 (95% CI: 0.76–0.82), 0.84 (0.81–0.86), 0.82 (0.79–0.84), and 0.81 (0.78–0.83)	None
	Landfeldt (2016) [100]		Cross-sectional study to estimate HRQoL in patients with DMD	770 patient-caregiver pairs (Germany, Italy, UK, US)	HUI (caregiver proxy-assessed)	The findings revealed a dramatic decrease in HUI-derived utilities ranging from 0.75 in early ambulatory patients to 0.15 non-ambulatory severely affected patients (*p* < 0.0001)	There was a discrepancy between HUI-derived utilities and the caregivers’ ratings of patients’ health and mental status across ambulatory classes (e.g., low utility estimates for patients perceived to be happy and in good health)
	Landfeldt (2015) [101]		Use of data from a cross-sectional study to develop and test the psychometric properties of the UK English version of DMDSAT	186 patient-caregiver pairs (UK)	Mapping from DMDSAT to HUI through regression analysis	The mean loss in patient utilities associated with a one-point change in DMDSAT total score was 9.5%. Spearman’s correlation between predicted and observed utility was 0.85 (*p* < 0.0001)	DMDSAT maps well onto health economic outcomes in this population
HST3 (20/7/2016) Ataluren/Duchenne muscular dystrophy	Landfeldt (2016) [99]		Cross-sectional study to estimate the caregiver burden associated with DMD	770 caregivers (Germany, Italy, UK, US)	EQ-5D-3L and VAS (0–1) collected from caregivers	Mean EQ-5D utility ranged between 0.85 (95% CI 0.82–0.88) and 0.77 (0.74–0.80) across ambulatory classes and 0.88 (0.85–0.90) and 0.57 (0.39–0.74) across caregivers’ rating of patients’ health and mental status. Mean VAS score was 0.74 (0.73–0.75).	Given how insensitive the EQ-5D appears to be to capture impairment in HRQL in many conditions, the considerable loss in utility observed in this study is both surprising and noteworthy, and captures the exquisite stresses associated with caring for a child with a chronically disabling and progressive condition. The small change in utility and VAS scores across ambulatory classes indicates that caregivers may find ways to learn to cope with the disease and the increasing levels of disability and morbidity associated with the progression of DMD and adjust their perception of their own HRQL over time.
	Landfeldt (2018) [98]		Cross-sectional study to assess the psychometric properties of the UK and US version of PedsQL NMM	278 patients (UK and US)	HUI3	Mean utility (SD): 0.36 (0.28)	None
	Landfeldt (2020) [97]		Delphi panel study to investigate consensus regarding utilities for ambulatory and non-ambulatory patients treated with ataluren plus best supportive care vs best supportive care alone	6 neuromuscular experts (Sweden)	HUI and VAS	Utility differences between treatments were 0.31 for ambulatory patients, and 0.15 to 0.18 for non-ambulatory patients, respectively. The corresponding VAS differences were 12 and 13.	Given the generic nature of the scale, not all questions in the HUI were found to be directly applicable to all patients with DMD. One potential reason for the observed inconsistencies among panelists, in addition to perceived differences in experiences of the clinical benefits of ataluren, is the heterogeneous presentation of DMD.
HST3 (20/7/2016) Ataluren/Duchenne muscular dystrophy		Magnetta (2018) [112]	Cost-effectiveness analysis of destination ventricular assist device therapy (DT-VAD)	Hypothetical cohort of patients with DMD and advanced heart failure	Utilities were derived from adult literature according to NYHA class of heart failure and modified for DMD by adjusting downward by a factor of 0.817 based on data comparing QOL in adolescents with DMD to those without DMD	Utilities: NYHA class I: 0.699; NYHA class II: 0.630; NYHA class III: 0.550; NYHA class IV: 0.435	None
		Pangalila (2012) [123]	Cross-sectional study to describe subjective caregiver burden of parents of adults with DMD	80 parents of 57 adult patients (Netherlands)	EQ-5D-3L completed by parents and patients; CarerQol VAS (parents)	EQ-5D value (mean ± SD): patients: 0.44 ± 0.13; parents: 0.87 ± 0.17. CarerQol VAS (mean ± SD): 7.4 ± 1.5	None

*AE-QoL* Angioedema Quality of Life, *AFD* Anderson Fabry Disease, *AHUS* atypical haemolytic uremic syndrome, *ASUI* Asthma Symptom Utility Index, *BPI-SF* Brief Pain Inventory Short Form, *CARE-PF* Canadian Registry for Pulmonary Fibrosis, *CF* cystic fibrosis, *CFRSD–CRISS* Cystic Fibrosis Respiratory Symptom Diary—Chronic Respiratory Infection Symptom Score, *CFQ-R* Cystic Fibrosis Questionnaire-Revised, *CI* confidence interval, *DLCO* diffusing capacity of carbon monoxide, *DMD* Duchenne Muscular Dystrophy, *DMDSAT* Duchenne Muscular Dystrophy Functional Ability Self-Assessment, *EQ-5D-3L* EuroQol-5 Dimension-3 Level, *EQ-5D-5L* EuroQol-5 Dimension-5 Level, *EQ-VAS* EuroQol-Visual Analogue Scale, *EQ-5D-Y* EuroQol-5 Dimension-Youth, *ERT* enzyme replacement therapy, *FEV1* forced expiratory volume in 1 s, *FPHPQ* Fabry-specific Paediatric Health and Pain Questionnaire, *FVC* forced vital capacity, *GD (1)* Gaucher’s Disease (type 1), *HAE* hereditary angioedema, *HAE-BOIS* Hereditary Angioedema Burden of Illness Study, *hATTR-PN* hereditary transthyretin amyloid polyneuropathy, *HRQoL* health-related quality of life, *HST* highly specialized technology, *HUI* health utility index, *ILD* interstitial lung disease, *IPF* idiopathic pulmonary fibrosis, *IRD* inherited retinal disease, *MID* minimum importance difference, *MPS* mucopolysaccharidosis, *3-MSCT* 3-min-stair-climb test, *6-MWT* 6-min walking test, *NGF* National Gaucher Foundation, *NYHA* New York Heart Association, *PBC* primary biliary cholangitis/cirrhosis, *PedsQL (NMM)* Paediatric Quality of Life Inventory 3.0 (Neuromuscular Module), *PE(s)* pulmonary exacerbation(s), *PSC* primary sclerosis cholangitis, *QWB* quality of well-being, *RDs* rare diseases, *SF-6D* Short Form-6 Dimension, *SG* standard gamble, *SMA* spinal muscular atrophy, *TA* technology appraisal, *TTO* time trade-off, *UK* United Kingdom, *US* United States, *VAS* visual analogue scale, *XLH* X-linked hypophosphatemia

**Table 3:** Synthesis of included studies (*n* = 112^a^)

	*N*	%
Disease		
Paediatric-onset hypophosphatasia	0	0.0
Atypical haemolytic uremic syndrome	3	2.7
Fabry disease	9	8.0
Hereditary transthyretin amyloidosis	3	2.7
Inherited retinal dystrophies	3	2.7
Cystic fibrosis (including pulmonary exacerbations)	36	32.1
Mucopolysaccharidosis type IVa	4	3.6
X-linked hypophosphatemia	1	0.9
Adenosine deaminase deficiency–severe combined immunodeficiency	1	0.9
Spinal muscular atrophy	5	4.5
Severe refractory eosinophilic asthma	1	0.9
Gaucher disease (type 1)	7	6.3
Limbal stem cell deficiency after eye burns	1	0.9
Hereditary angioedema	5	4.5
Primary biliary cholangitis	6	5.4
Waldenstrom’s macroglobulinemia	1	0.9
Idiopathic pulmonary fibrosis	16	14.3
Duchenne muscular dystrophy	10	8.9
Neuronal ceroid lipofuscinosis type 2	0	0.0
Location		
Multiple countries	27	24.1
US	22	19.6
UK	23	20.5
Netherlands	8	7.1
Sweden	6	5.4
Other countries	24	21.4
Not specified	2	1.8
Publication time		
Before 2000	14	12.5
2001–2010	20	17.9
2011–2020	78	69.6
Study design		
Clinical trial	19	17.0
Longitudinal/cohort/real-world study	33	29.5
Cross-sectional study	46	41.0
Cost-effectiveness/-utility analysis	14	12.5
Study primary outcome		
Clinical measures (QoL/utility as secondary outcome)	22	19.6
QoL	30	26.8
QoL and economic burden	12	10.7
Health state utility	15	13.4
Questionnaire(s) validation/feasibility/responsiveness/correlation	16	14.3
Cost-effectiveness/utility	14	12.5
Caregiver burden	3	2.7
Technique to estimate utility values^b^		
Standard gamble	11	9.8
Time trade-off	7	6.3
Risk–risk trade-off	1	0.9
Visual analogue scale (alone, not together EQ-5D)	10	8.9
EQ-5D (either 3L or 5L)	68	60.7
EQ-5D-Y	4	3.6
HUI2/3	14	12.5
QWB	11	9.8
15D	3	2.7
SF-6D	2	1.8
Disease-specific preference-based measures (i.e., ASUI)	1	0.9
CarerQoL-7D/-VAS	3	2.7
Mapping	3	2.7
Author’s original approach	3	2.7
Respondents^b^		
Patients	96	85.7
Caregivers/parents (proxy-reporting on patient’s status)	11	9.8
Caregivers/parents (reporting on their own status)	12	10.7
Clinicians/paediatricians/medical students	6	5.4
General public (healthy or with other conditions)	5	4.5
Use of case study/case history/vignettes		
Yes	9	8.0
No	103	92.0
Sample size		
< 100	47	42.0
100–300	30	26.8
> 300	28	25.0
Not specified	7	6.2
Methodological considerations on utility estimation		
Yes	57	50.9
No	55	49.1

*ASUI* Asthma Symptom Utility Index, *EQ-5D* EuroQol-5 Dimension, *EQ-5D-Y* EuroQol-5 Dimension-Youth, *HUI* Health Utility Index, *QoL* quality of life, *QWB* Quality of Well-being, *SF-6D* Short-Form 6-Dimension, *VAS* visual analogue scale

^a^One study (Wyatt 2012) addressed two diseases

^b^Multiple categories allowed

**Fig. 3:**
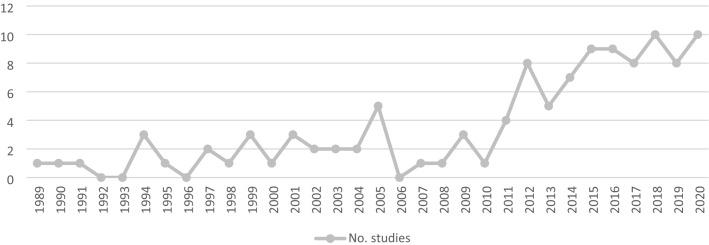
Number of studies by publication year

In terms of study location, 27 (24.1%) were international studies involving two or more countries. Among the single-country studies, 23 studies (20.5%) were conducted in the UK, 22 (19.6%) in the US, eight (7.1%) in the Netherlands, six (5.4%) in Sweden, five (4.5%) in Canada, three (2.7%) in Australia, Finland and Germany respectively, two (1.8%) in France, Spain and Japan respectively, and one (0.9%) in Switzerland, Ireland, Bulgaria, Portugal respectively; in two cases (1.8%), the study location was not specified. The great majority of studies (*n* = 78, 70%) were published after 2010 (Fig. 3). Over half of the studies (*n* = 64, 57.1%) were aiming to evaluate QoL using preference-based questionnaires that (involuntarily) also allowed to derive HSUVs; of these studies, 22 (19.6%) were clinical studies using QoL as secondary endpoint, 30 (26.8%) were primarily aiming to estimate QoL, and 12 (10.7%) were assessing QoL and economic burden together. Only 15 (13.4%) explicitly aimed to estimate HSUVs. The remaining aimed to validate existing questionnaires in a specific patient population (*n* = 16, 14.3%), to calculate the cost-effectiveness of treatments (*n* = 14, 12.5%) or to quantify caregiver burden (*n* = 3, 2.7%).

The majority of studies (*n* = 72, 64.3%), all published from 2000 onward, used one of the EuroQol instrument versions (i.e., EQ-5D-3L, EQ-5D-5L or EQ-5D-Y) to estimate HSUVs. The QWB scale and HUI 2/3 were chosen as preference-based instruments by 11 (9.8%) and 14 (12.5%) studies respectively, although the former was never used after 2005, while the latter was more equally distributed over time. Cumulatively, 97 studies (86.6%) used at least one preference-based instrument. Direct utility estimation methods were less frequently adopted: 11 (9.8%) studies used SG, ten (8.9%) used visual analogue scale, seven (6.3%) used TTO, and one (0.9%) the risk–risk trade-off, for a total of 14 studies (12.5%) using at least one of these approaches. In three studies (2.7%), caregiver utility was estimated through the Carer-QoL-7D/-VAS. In three studies (2.7%) HSUVs were mapped from non-preference-based measures, while in other three (2.7%) the authors developed an original approach to obtain HSUVs, such as adjusting data from the literature.

The great majority of studies (*n* = 96, 85.7%) surveyed patients to obtain HSUVs; when the patient was a child, the parent/caregiver support or proxy-reporting was used by 11 (9.8%) studies. Caregivers also reported on their own health in 12 studies (10.7%) which aimed at quantifying the caregiver’s burden, exclusively or in addition to patient’s burden. Finally, very few studies recruited other types of responders such as clinical experts (*n* = 6, 5.4%) or the general public (*n* = 5, 4.5%) to derive HSUVs using proxy reporting (e.g., paediatricians valuing their patient’s health) or ‘vignettes’. In particular, the use of health state descriptions or case studies/histories (‘vignettes’) was reported by 9 studies only (8.0%). The sample size was classified as small (< 100 participants) in 42% of the studies (*n* = 47).

### Synthesis of authors’ methodological considerations

Overall, 57 (out of 111) studies retrieved from the literature discussed the pros/cons of the techniques adopted to estimate HSUVs. Their authors’ methodological considerations are reported verbatim in Table 2. Of these, 22 (38.6%) were studies assessing QoL through preference-based PROMs (with or without economic burden), 14 (24.6%) estimating HSUVs as specific objective, and 13 (22.8%) assessing the validity or feasibility of questionnaires. This section presents a synthesis of these authors’ statements for each rare disease covered by the literature review, with the exception of three (i.e., atypical haemolytic uremic syndrome, severe combined immunodeficiency, and Waldenstrom’s macroglobulinaemia), for which no studies commented on the methods to estimate HSUVs.

#### Cystic fibrosis

Of the 22 studies providing methodological considerations, half (*n* = 11) commented on the use of the EQ-5D questionnaires (i.e., EQ-5D-3L/-5L and EQ-5D-Y) to estimate HSUVs. The overall evaluation was positive in seven studies [49, 50, 55, 59, 66, 67, 75], where EQ-5D was described as a valid, easy, and cheap generic instrument reflecting disease progression and severity, as well as allowing cross-disease comparisons and country-specific cost-effectiveness analyses. Conversely, four studies [48, 57, 81, 139] revealed its high ceiling effects and poor sensitivity in capturing symptoms (particularly, the self-care domain was considered irrelevant), and encouraged the use of alternative generic instruments (e.g., HUI3 and SF-6D), or, preferably, the development of a disease-specific preference-based measure (also including a respiratory domain).

The remaining 11 studies advised on alternative techniques to derive HSUVs. The judgment on QWB was mixed, with some studies [69, 74, 116] highlighting its low correlation with other measures of physical and emotional functioning, poor sensitivity in detecting changes in functional domains, or the limitations of its scoring system, while others [116, 121, 122] arguing that it showed responsiveness and validity in tracking changes in patients’ health, even during pulmonary exacerbations, and significantly correlated with measures of performance and pulmonary function. Similarly, the HUI was found to include relevant dimensions for cystic fibrosis patients, such as hearing and fertility [91, 153], but failing to discriminate between severity levels, and too difficult for paediatric patients to complete, thus requiring parental proxy-reporting [125, 153]. The only study [77] using CarerQol-7D showed that this instrument is easy and presents some external validity. Finally, two studies adopting direct methods to estimate HSUVs reached opposite conclusions, as one [62] argued that SG tends to produce higher values than other techniques, whereas the other [148] argued that, assuming individuals are neutral to risk, it is likely to underestimate utilities in patients that are keener to accept risk, such as those waiting for lung transplantation. In one study [116], the small number of patients (*n* = 20) limited to investigate the responsiveness, validity and reproducibility of the instruments adopted.

#### Idiopathic pulmonary fibrosis

The judgment on EQ-5D was overall positive, with several studies [58, 85, 142, 143] reporting that the instrument was sensitive to short-term health status changes and comparable to disease-specific questionnaires (e.g., K-BILD), and also allowed comparison with different lung diseases and the general population. However, EQ-5D also presented a significant ceiling effect in mild disease and may not fully capture disease-specific issues [58, 143]. Similarly, the QWB was considered not sensitive enough in distinguishing between heterogeneous disease’s manifestations and not correlated with physiological measures (e.g., dyspnoea scale) [65, 145].

#### Duchenne muscular dystrophy

The authors’ considerations on preference-based PROMs were generally negative since both EQ-5D and HUI lacked sufficient sensitivity to be used in mobility impaired patients [60]. In particular, the EQ-5D has no consideration for mobility beyond walking, thus underestimating the utility reduction for DMD children [60] and was considered insensitive in this condition [99]. The HUI did not correlate with caregiver’s rating of the patient’s health [100], and given its generic nature, was not considered to be applicable to patients with heterogeneous DMD presentations [97]. Conversely, one study [101] reported that the Duchenne Muscular Dystrophy Functional Ability Self-Assessment (DMDSAT) mapped well onto HUI in this patient population.

#### Other diseases

In Fabry disease, three studies highlighted the limitations of the use of EQ-5D-3L, such as its inability to detect small health changes, by having only three levels per domain [51], and to capture real health state changes, rather than patient’s perceptions of their health state [88]. Moreover, because of disease’s rarity, it was necessary to pool questionnaires in different languages to obtain a sufficiently large sample [128].

Conversely, two studies performed in hereditary transthyretin amyloidosis argued that EQ-5D showed validity and appropriateness, in spite of difficulties due to disease rarity and small samples [89, 119].

The three studies addressing inherited retinal dystrophies (IRDs) used very different approaches. One study [61] argued that TTO is easier to understand by patients compared to SG, and that the latter also overestimates risk aversion, since for each visual acuity stage a lower percentage of patients was willing to risk death (SG) than trade time of life (TTO) to return to perfect vision. The second study [70] reported that EQ-5D showed significant ceiling effects and may not be suitable for patients with genetic eye diseases, despite presenting the advantage of allowing comparison among alternative conditions. The third [107] reported that, due to the ultra-rarity of the disease, the study could not recruit patients and instead developed vignettes to describe IRDs health states, which were subsequently evaluated by six retina specialists using EQ-5D-5L. The authors’ judgment on this approach was overall positive, since descriptions of IRDs may not be easily understood by the public and the qualitative research that informed vignettes to be valued by clinical experts supported the face validity of the HSUVs obtained.

Among the studies performed in mucopolysaccharidosis type Iva (also known as Morquio A), one [86] reported that EQ-5D-5L was not sensitive enough in assessing pain severity if compared with the Brief Pain Inventory, while another [126] admitted having used it, because it was recommended by international guidelines. A further study [87] showed that medical experts (*n* = 6) and students (*n* = 93), used as surrogate respondents for patients who are often children with intellectual disability, reported comparable results using direct techniques. The authors also argued that indirect methods (e.g., EQ-5D) could be more practical with larger samples, although their validity in mucopolysaccharidoses was not demonstrated yet.

The only study [80] addressing X-linked hypophosphatemia reported that EQ-5D-5L may not capture disease-specific problems and that preferences in patients with rare bone diseases may not be aligned with those of the general population, which informed the instrument’s valuation system.

In spinal muscular atrophy (SMA), one study [56] discussed the pros and cons of using HUI3, which measures the ability to carry out daily activities (e.g., dressing) that are relevant for physically impaired SMA patients, but also dimensions such as seeing and hearing that are not impacted by the disease. A further study [106] highlighted the limitations of using vignettes to develop SMA case histories and asking clinical experts to value them using EQ-5D-Y. Indeed, standard descriptions of SMA states do not capture the heterogeneous manifestations of the disease, and the accuracy of HSUVs depends on the validity or accuracy of such descriptions and their proxy evaluation. However, the EQ-5D-Y has not been validated in children as young as patients with infantile-onset SMA and using physicians for proxy reporting allow to avoid some bias that parental judgment may introduce. Finally, a third study [156] highlighted the limitations of applying an existing mapping algorithm to SMA patients.

The only study dealing with severe refractory eosinophilic asthma [108] used both the generic EQ-5D and the disease-specific Asthma Symptom Utility Index (ASUI), commenting that the latter may show a floor effect for patients with severe asthma.

In Gaucher disease (GD) of type 1, the four studies providing methodological considerations used direct techniques to estimate HSUVs. The first [68] revealed that, among the techniques used for valuing vignettes by healthy volunteers, chronically ill patients and GD patients, the risk–risk trade-off yielded significantly lower utilities than SG and TTO and showed the poorest test–retest reliability. The second study [79] showed that the WTP approach (used to determine the maximum amount healthy volunteers were willing to pay to have an insurance scheme covering the treatment cost) produced results aligned with SG (used to determine the maximum risk of death they were willing to bear to avoid living with GD), and may be well-suited to elicit preferences for chronic conditions where it is unrealistic to trade-off life duration (as in TTO) or risk of immediate death (as in SG). The third study [84] pointed out the limitations of using health state descriptions that were developed without direct inputs from GD patients and valued by the general population. The fourth study [104] reported that using different search procedures (i.e., the method—titration and “ping-pong”—used to find the point of indifference) significantly impacted the HSUVs estimated by healthy volunteers.

The only study providing methodological considerations in primary biliary cholangitis suggested that HUI performed well since was able to discriminate between patients with mild and severe disease [154].

The only study [138] addressing limbal stem cell deficiency (LSCD) pointed out the limitations of using vignettes to be valued by members of the general public, since some participants failed to correctly interpret LSCD descriptions, and there was limited overlap between the EQ-5D-3L dimensions and LSCD vignettes, despite the inclusion of the vision bolt-on. However, given the rarity of the condition and the small sample achievable, deriving utilities directly from patients would have provided unreliable estimates.

In hereditary angioedema, a study mapping the HAE-BOIS survey items onto EQ-5D-3L reported that the items selected for manual cross-walking were conceptually overlapping with EQ-5D dimensions [53]. The two studies by Nordenfelt et al. [117, 118] reported that EQ-5D-5L is suitable to assess QoL in this condition, and also includes the pain element, as opposed to disease-specific instruments, such as AE-QoL.

### Comparison between NICE reports and published studies

Table 4 summarizes the approaches used to derive HSUVs included in the manufacturer NICE submissions (as reported in TA/HST guidance documents, *n* = 22) with the existing published studies (*n* = 111) that estimated HSUVs for each individual rare disease considered in this review, irrespective of the publication date and the consequent availability (or not) to manufacturers for developing their economic models.

**Table 4:** Comparison between manufacturer and literature approaches to estimate HSUVs in RDs

Disease	Manufacturers (TA/HST)	Published literature (number of studies)
Paediatric-onset hypophosphatasia	Vignettes valued using EQ-5D-5L by clinical experts (*HST6*)	None
Atypical haemolytic uremic syndrome	EQ-5D collected from patients (*HST1*)	EQ-5D collected from patients (*n* = 3)
Fabry disease	DCE with the public (*HST4*)	EQ-5D collected from patients (*n* = 8) EQ-5D and SF-36 (SF-6D) collected from patients (*n* = 1)
Hereditary transthyretin amyloidosis	EQ-5D values from the literature (*HST9*) EQ-5D-5L mapped onto EQ-5D-3L collected from patients (*HST10*)	EQ-5D collected from patients (*n* = 3)
Inherited retinal dystrophies	Vignettes valued using HUI3 and EQ-5D by clinical experts (*HST11*)	SG and TTO performed by patients (*n* = 1) EQ-5D-3L collected from patients (*n* = 1) Vignettes valued using HUI3 and EQ-5D by clinical experts (*n* = 1)
Cystic fibrosis	CFQ mapped onto EQ-5D using published algorithm (*TA276*) HUI2 collected from patients + utilities from the literature (*TA266*) EQ-5D collected from patients + utilities from the literature (*TA398*)	EQ-5D-3L collected from patients or caregivers (*n* = 9) EQ-5D-5L collected from patients or caregivers (*n* = 5) QWB collected from patients or caregivers (*n* = 9) CFQ-R mapped onto EQ-5D-3L (*n* = 1) Direct techniques (SG, TTO, or VAS) performed by patients (*n* = 3) HUI2/3 collected from patients, caregivers, or clinicians (*n* = 5) EQ-5D-Y collected from patients (*n* = 1) CarerQoL-7D + VAS collected from caregivers (*n* = 1) Direct + indirect techniques performed by patients (*n* = 2)
Mucopolysaccharidosis type IVa	EQ-5D-5L collected from patients + utilities from the literature (*HST2*)	EQ-5D-5L collected from patients (*n* = 2) Vignettes valued using direct techniques (SG, TTO, VAS) by clinical experts and students (*n* = 1) EQ-5D-3L collected from patients and caregivers (*n* = 1)
X-linked hypophosphatemia	Vignettes valued using EQ-5D-5L by clinical experts (*HST8*)	EQ-5D-5L collected from patients (*n* = 1)
Severe combined immunodeficiency	Utilities from the literature (*HST7*)	Clinical experts’ opinion (*n* = 1)
Severe refractory eosinophilic asthma	EQ-5D collected from patients (*TA431*)	EQ-5D and ASUI collected from patients (*n* = 1)
Limbal stem cell deficiency	Vignettes valued using SG by members of the public (*TA467*)	Vignettes valued using EQ-5D-3L + V and SG by members of the public (*n* = 1)
Primary biliary cholangitis	Utilities from the literature (*TA443*)	15D collected from patients (*n* = 3) EQ-5D collected from patients (*n* = 2)
Spinal muscular atrophy	Clinical experts’ opinion + utilities from the literature (*TA588*)	HUI3 collected from patients or caregivers (*n* = 1) EQ-5D-Y collected from patients or caregivers + CarerQoL-7D collected from caregivers (*n* = 1) Vignettes valued using EQ-5D-Y by clinical experts (*n* = 1) EQ-5D collected from caregivers (*n* = 1) Utilities from the literature + original author’s approach to estimate caregiver’s utilities (*n* = 1)
Gaucher disease (type 1)	SF-36 mapped onto EQ-5D using a published algorithm (*HST5*)	Vignettes valued using various direct methods (SG, TTO, RRTO, VAS, WTP) by healthy volunteers, chronically ill patients, or GD patients (*n* = 4) EQ-5D-3L collected from patients (*n* = 2) EQ-5D and SF-6D collected from patients (*n* = 1)
Hereditary angioedema	EQ-5D-5L values from the literature (*TA606*)	Mapping from HAE-BOIS survey items onto EQ-5D-3L (*n* = 1) EQ-5D-3L collected from patients (*n* = 1) EQ-5D-5L collected from patients (*n* = 2) EQ-5D-Y collected from patients (*n* = 1)
Waldenstrom’s macroglobulinemia	EQ-5D-5L collected from patients + utilities from the literature (*TA491*)	EQ-5D-5L collected from patients (*n* = 1)
Idiopathic pulmonary fibrosis	EQ-5D collected from patients (*TA379*)	EQ-5D (3L or 5L) collected from patients (*n* = 14) QWB collected from patients (*n* = 1) QWB collected from patients and healthy subjects (*n* = 1) HUI2 collected from patients (*n* = 1)
Duchenne muscular dystrophy	Utilities from the literature (*HST3*)	EQ-5D-Y and HUI2/3 collected from patients and caregivers (*n* = 1) EQ-5D collected from patients or caregivers (*n* = 1) HUI collected from patients and EQ-5D from caregivers (*n* = 1) HUI collected from caregivers (*n* = 1) DMDSAT mapped into HUI (*n* = 1) EQ-5D-3L and VAS collected from caregivers (*n* = 1) HUI3 collected from patients (*n* = 1) HUI and VAS collected from clinical experts within a Delphi study (*n* = 1) EQ-5D-3L collected from patients and caregivers + CarerQol VAS collected from caregivers (*n* = 1) Authors’ original approach (*n* = 1)
Neuronal ceroid lipofuscinosis type 2	Vignettes valued using EQ-5D-5L by clinical experts (*HST12*)	None

*ASUI* Asthma Symptom Utility Index, *CFQ* Cystic Fibrosis Questionnaire, *DMDSAT* Duchenne Muscular Dystrophy Functional Ability Self-Assessment, *EQ-5D* EuroQol 5-Dimension, *EQ-5D-Y* EuroQol 5-Dimension Youth, *HAE-BOIS* Hereditary Angioedema Burden of Illness Study, *HST* highly specialized technology, *HSUV* health state utility values, *HUI* health utility index, highly specialized technology; *QWB* Quality of Well-being, *RDs* rare diseases, *RRTO* risk–risk trade-off, *SF-36* Short-Form 36, *SF-6D* Short-Form 6-Dimension, *SG* standard gamble, *TA* technology appraisal, *TTO* time trade-off, *VAS* visual analogue scale, *WTP* willingness-to-pay

The use of ‘vignettes’ valued by non-patient populations (i.e., clinical experts or members of the public) was more frequent in NICE technology appraisals than in the literature (5/22, 22.7% vs. 9/112, 8.0%), as well as the use of ‘mapping’ from non-preference-based measures (1/22, 9.1% vs. 3/112, 2.7%). In 36.4% (8/22) of cases, the manufacturers used preference-based PROMs (i.e., EQ-5D and/or HUI) collected from patients (and/or caregivers), while this methodology (alone or in combinations with others) was adopted by 83.0% (93/112) of published studies. Moreover, the literature presented a much wider range of preference-based PROMs including also the generic QWB, SF-6D, and 15D, the CarerQol-7D (specifically for caregivers), and the disease-specific ASUI. The use of direct methods (e.g., SG, TTO, VAS, RRTO) was reported by one manufacturer submission only (1/22, 4.5%) versus 14 published studies (12.5%). One NICE document (4.5%) reported on a DCE performed with the general public, while this technique was not found in the literature reviewed. Finally, in ten NICE appraisals (45.5%) the manufacturers obtained utilities from the literature.

In relation to individual RDs, a high level of agreement in the methodological choices was observed in atypical haemolytic syndrome, where NICE technology appraisal (HST1) and the three studies retrieved from the literature [76, 103, 105] collected EQ-5D directly from patients, as well as in severe refractory eosinophilic asthma, where original utilities were estimated using EQ-5D in TA431, as in the study by Lloyd [108] with the addition of ASUI. Similarly, in Waldenstrom’s macroglobulinemia, both the NICE report (TA491) and published study [73] collected EQ-5D-5L from patients, as well as in idiopathic pulmonary fibrosis, where TA379 and 14 (out of 17) studies used EQ-5D (either 3L or 5L). In cystic fibrosis, the use of preference-based instruments (i.e., EQ-5D and HUI2) and mapping from CFQ was common between technology appraisals (TA266, TA276, TA398) and most of the studies retrieved (22 out of 36).

Conversely, a low agreement between the approaches used in the NICE report and the literature was found in relation to Fabry disease, where the former (HST4) performed a DCE with members of the general population, while the latter collected EQ-5D-3L in eight cases [51, 54, 88, 114, 115, 128, 131, 144] (and, in one study [152], also SF-36 to be valued using SF-6D). In addition, in X-linked hypophosphatemia, the technology appraisal (HST8) estimated HSUVs using vignettes valued by clinical experts through EQ-5D-5L, while in the only study retrieved [80] the same instrument was used to collect data directly from patients. In spinal muscular atrophy, the technology appraisal (TA588) relied on expert opinion and the literature to derive HSUVs, while a variety of approaches (including preference-based instruments collected from patients, caregivers, or clinical experts using vignettes) were presented by the five studies retrieved [56, 64, 106, 110, 156].

In Gaucher disease, the manufacturer’s approach (HST5) consisted in mapping SF-36 onto EQ-5D using a published algorithm; conversely, three published studies [71, 147, 152] collected preference-based measures from patients, while another four [68, 79, 84, 104] used vignettes to be valued using various direct techniques. In DMD, the manufacturer (HST3) derived utilities from the literature (because the algorithm available to convert PedsQL data collected in the clinical study onto EQ-5D utilities was developed in a healthy population), while several preference-based measures (i.e., EQ-5D, EQ-5D-Y, HUI2/3, CarerQol-7D) were used in eight (out of 10) published studies [60, 63, 97–99, 101, 102, 123].

### Degree of exploitation of the existing literature in manufacturers’ submissions

In Table 5, the methods chosen by manufacturers to obtain HSUVs are compared with: (1) the alternative techniques recommended by NICE ERG and/or Committee (excluding suggestions of minor adjustments that do not alter the main technique, as reported in Table 1) and (2) the techniques adopted in studies that were published at least one year before the TA/HST date (and, therefore, presumably available to manufacturers at the time of drafting their submission), together with a synthesis of study’s authors methodological considerations.

**Table 5:** Synthesis of techniques used by manufacturers, suggested by ERG/Committee, and used by published studies to estimate HSUVs

NICE’s reports				Published literature		
Report code	Drug	Manufacturer’s approach	ERG/Committee’s suggested alternative approach	Published studies (at least one year before NICE appraisal date)	Study’s approach	Study’s methodological considerations (synthesis)
Report date	Disease					
HST6	Asfotase alfa	EQ-5D-5L (vignettes based on 6MWT)	None			
2/8/2017	Paediatric-onset hypophosphatasia					
				None		None
HST1	Eculizumab	EQ-5D	None			
28/1/2015	Atypical haemolytic uremic syndrome					
				Legendre (2013) [103]	EQ-5D	None
HST4	Migalastat	DCE	Literature values (NS)			
22/2/2017	Fabry Disease					
				Beck (2004) [54]	EQ-5D-3L	None
				Hoffmann (2005) [88]	EQ-5D-3L	EQ-5D-3L has some limitations
				Mehta (2009) [114]	EQ-5D	None
				Miners (2002) [115]	EQ-5D	None
				Ramaswami (2012) [128]	EQ-5D	None
				Rombach (2013) [131]	EQ-5D-3L	None
				Tsuboi (2012) [144]	EQ-5D-3L	None
				Wyatt (2012) [152]	EQ-5D, SF-6D	None
HST9	Inotersen	EQ-5D literature values (Stewart 2017)	None			
22/5/2019	Hereditary transthyretin amyloidosis					
				Wixner (2014) [151]	EQ-5D	None
HST10	Patisiran	EQ-5D-5L (mapped to 3L)	None			
14/8/2019	Hereditary transthyretin amyloidosis					
				Wixner (2014) [151]	EQ-5D	None
HST11	Voretigene neparvovec	HUI3 and EQ-5D (vignettes)	TTO values from Rentz (2014)			
9/10/2019	Inherited retinal dystrophies					
				Brown (1999) [61]	SG, TTO	TTO is more understandable than SG by patients; SG overestimates risk aversion
				Davison (2017) [70]	EQ-5D-3L	EQ-5D displays significant ceiling effects, and its suitability for patients with genetic eye conditions has yet to be determined; however, it enables comparison across health conditions.
TA266	Mannitol dry powder	HUI2; literature values (NS)	EQ-5D			
28/11/2012	Cystic fibrosis					
				Anyanwu (2001) [50]	EQ-5D-3L	The EQ-5D-3L is responsive in changes of quality-of-life
				Busschbach (1994) [62]	SG, TTO, EQ-VAS	SG produces the highest values, EQ-VAS the lowest
				Czyzewski (1994) [69]	QWB	Correlations between the QWB and other indicators of physical and psychosocial functioning are low. The use of QWB in paediatric CF patients is questionable.
				Dunlevy (1994) [74]	QWB	The lack of improvement in QWB may be related to the relative weight of the symptom/problem subscale
				Eidt-Koch (2009) [75]	EQ-5D-Y	The EQ-5D-Y can be considered a cross-sectional valid generic instrument reflecting differences in health according to the progression of the life-long chronic disease CF
				Fitzgerald (2005) [78]	QWB	None
				Groen (2004) [82]	EQ-5D	None
				Janse (2005) [92]	HUI3	None
				Janse (2008) [91]	HUI3	The attributes of the HUI3 (e.g., hearing) match well with major complaints of CF patients
				Johnson (2000) [93]	EQ-5D-3L	None
				Munzenberger (1999) [116]	QWB	The scale is a responsive measure of quality-of-life outcome of pulmonary exacerbation. However, the scale is a generic instrument and may not be sensitive enough to detect changes in function domains in patients with CF
				Orenstein (1989) [122]	QWB	The QWB scale significantly correlates with measures of performance and pulmonary function and has substantial validity as an outcome measure in CF patients
				Orenstein (1990) [121]	QWB	The QWB can track changes in general well-being in CF patients over a brief time and detect changes associated with pulmonary exacerbations and its treatment and allows for comparison with other conditions
				Orenstein (1991) [120]	QWB	None
				Petrou (2009) [125]	HUI3	The comprehension level for the HUI is somewhat high for a paediatric sample
				Ramsey (1995) [129]	SG	None
				Selvadurai (2002) [134]	QWB	None
				Suri (2001) [141]	QWB	None
				Vasiliadis (2005) [148]	SG	SG might underestimate the utility associated while waiting for lung transplantation
				Yi (2003) [153]	HUI2, VAS, SG, TTO	The HUI2 does not appear to discriminate well between levels of health. However, because the issue of fertility is important in CF, an advantage of the HUI2 is that is addresses fertility.
TA276	Colistimethate sodium and tobramycin dry powders for inhalation	Mapping CFQ onto EQ-5D using published coefficients (Eidt-Koch 2009)	Utilities from Bradley (2010)	a		
27/3/2013	Pseudomonas lung infection in cystic fibrosis					
				Dewitt (2012) [72]	HUI2/3, VAS	None
				Santana (2012) [133]	HUI3	None
TA398	Lumacaftor-ivacaftor	EQ-5D; literature values (Whiting 2014)	None	a		
27/07/2016	Cystic fibrosis					
				Acaster (2015) [48]	Mapping CFQ-R onto EQ-5D-3L	There is a tendency of over-/under-prediction in negative/positive states, and respiratory domain is not a significant predictor of EQ-5D utility
				Angelis (2015) [49]	EQ-5D-5L	The EQ-5D-5L can be considered a cross-sectional valid generic health outcome measure reflecting the progression of CF
				Bradley (2013) [59]	EQ-5D-3L	The EQ-5D correlates strongly with disease-specific domains of the CFQ-R, and allows for comparisons with other respiratory populations
				Chevreul (2015) [67]	EQ-5D-5L	The EQ-5D is a generic tool that enable utility calculations; its use in children and parent-proxy ratings have also been shown to be both feasible and valid
				Iskrov (2015) [90]	EQ-5D-3L	None
				Singer (2015) [137]	EQ-5D, SG, VAS	None
				Whiting (2014) [150]	EQ-5D	None
HST2	Elosulfase alfa	EQ-5D-5L; literature values (NS)	None			
16/12/2015	Mucopolysaccharidosis type IVa					
				Hendriksz (2014) [86]	EQ-5D-5L	The EQ-5D pain/discomfort domain scores did not follow the pain severity scores obtained by the BPI-SF
				Hisashige (2011) [87]	VAS, SG, TTO (vignettes)	Patients of MPSs were not used as the respondents, since they are often children with mental retardation, who would have difficulty in evaluating utility of their own health states
HST8	Burosumab	EQ-5D-5L (vignettes)	None			
10/10/2018	X-linked hypophosphatemia					
				Forestier-Zhang (2016) [80]	EQ-5D-5L	The EQ-5D-5 L is a generic measure and may not capture specific problems for this patient group; also, the utility score is derived from the preferences of the general population and the preferences for patients with rare bone diseases may differ.
HST7	Strimvelis	Literature values (NS)	None			
7/2/2018	Adenosine deaminase deficiency-severe combined immunodeficiency					
				None		
TA588	Nusinersen	Clinical advice; literature values (NS)	None			
24/7/2019	Spinal muscular atrophy					
				López-Bastida (2017) [110]	EQ-5D	None
TA431	Mepolizumab	EQ-5D	None			
25/1/2017	Severe refractory eosinophilic asthma					
				Lloyd (2007) [108]	EQ-5D; ASUI	There is some evidence of floor effect on ASUI in its ability to capture the impact of exacerbations
HST5	Eliglustat	Mapping SF-36 onto EQ-5D	None			
28/6/2017	Gaucher disease (type 1)					
				Clarke (1997) [68]	SG; TTO, RRTO (vignettes)	The RRTO produces significantly lower utilities than the other two preference assessment methods and has the poorest test–retest reliability
				Deegan (2011) [71]	EQ-5D-3L	None
				van Dussen (2014) [147]	EQ-5D	None
				Flowers (1997) [79]	SG; WTP (vignettes)	The WTP method may be particularly well-suited to measure preferences for conditions where it is unrealistic to trade-off life expectancy or to take a risk of immediate death
				Lenert (1998) [104]	TTO; SG; VAS (vignettes)	The search procedure (titration or “ping-pong”) strongly influences the results of preference-assessment experiments
				Wyatt (2012) [152]	EQ-5D; SF-6D	None
TA467	Holoclar	SG	None			
16/8/2017	Limbal stem cell deficiency after eye burns					
				None		
TA606	Lanadelumab	Literature values (Nordenfelt 2014)	None			
16/10/2019	Hereditary angioedema					
				Aygören-Pürsün (2016) [53]	Mapping HAE-BOIS-Europe survey items onto EQ-5D-3L	The HAE-BOIS-Europe survey items selected for the manual cross-walking to the EQ-5D appear to be sufficiently comparable conceptually
				Lumry (2018) [111]	EQ-5D-3L	None
				Nordenfelt (2014) [117]	EQ-5D-5L	In the absence of a validated disease-specific instrument, the EQ-5D-5L can be used to measure the HAE disease impact
				Nordenfelt (2017) [118]	EQ-5D-5L	The EQ-5D contains the pain aspect, whereas disease-specific instruments such as the AE-QoL do not
TA443	Obeticholic acid	Literature values (Younossi 2001; Wright 2006)	None			
26/4/2017	Primary biliary cholangitis					
				Aberg (2011) [47]	15D	None
				Aberg (2012) [46]	15D	None
				Haapamaki (2015) [83]	15D	None
				Longworth (2003) [109]	EQ-5D	None
				Rice (2020) [130]	EQ-5D-5L	None
				Younossi (2001) [154]	HUI2	The HUI performs well
TA491	Ibrutinib	EQ-5D-5L, literature values (Beusterien 2010; Tolley 2013)	None			
22/11/2017	Waldenstrom’s macroglobulinaemia					
				None		
TA379	Nintedanib	EQ-5D	None			
27/1/2016	Idiopathic pulmonary fibrosis					
				Chang (1999) [65]	QWB	This generic questionnaire may be less successful in distinguishing between disease severity levels
				Han (2013) [85]	EQ-5D	EQ-5D may be more sensitive to short-term QoL changes than the SF-36 in idiopathic pulmonary fibrosis
				King (2011) [94]	EQ-5D	None
				Martinez (2014) [113]	EQ-5D	None
				Shulgina (2013) [135]	EQ-5D-3L	None
				Tzanakis (2005) [145]	QWB	There is no correlation between QWB and physiological parameters
				Zisman (2010) [155]	EQ-5D	None
HST3	Ataluren	Literature values (Landfeldt et al. 2014)	None			
20/7/2016	Duchenne muscular dystrophy					
				Landfeldt (2014) [102]	HUI; EQ-5D	None
				Landfeldt (2015) [101]	Mapping DMDSAT onto HUI	DMDSAT maps well onto health economic outcomes in this population
				Pangalila (2012) [123]	EQ-5D-3L; CarerQol VAS	None
HST12	Cerliponase alfa	EQ-5D-5L (vignettes)	None			
27/11/2019	Neuronal ceroid lipofuscinosis type 2					
				None		

*6MWT* 6 min walk test, *ASUI* Asthma Symptom Utility Index, *BPI-SF* Brief Pain Inventory Short Form, *CF* cystic fibrosis, *CFQ* cystic fibrosis questionnaire, *DMDSAT* Duchenne Muscular Dystrophy Functional Ability Self-Assessment, *EQ-5D* EuroQol-5 Dimension, *EQ-5D-Y* EuroQol-5 Dimension-Youth, *ERG* Evidence Review Group, *HAE* hereditary angioedema, *HAE-BOIS* Hereditary Angioedema Burden of Illness Study, *HST* highly specialized technology, *HUI* Health Utility Index, *NICE* National Institute for Health and Care Excellence, *NS* not specified, *QWB* quality of well-being, *RRTO* risk–risk trade-off, *SG* standard gamble, *VAS* visual analogue scale, *TA* technology appraisal, *TTO* time trade-off, *WTP* willingness-to-pay

^a^Additional studies compared to the previous report in the same RD

Overall, 72 studies (out of 112, 64.3%) were assumed to be available to manufacturers at the time of their drug submissions. In detail, five studies [75, 102, 117, 150, 154] included in our literature review were explicitly mentioned by the manufacturers as a source to obtain utilities that were not collected in their clinical studies (HST3, TA398, TA443, TA606), or to derive published coefficients [75] to perform a mapping exercise (TA276). In three cases [75, 117, 154], the studies provided positive recommendations regarding the instruments used (i.e., EQ-5D-Y, EQ-5D-5L and HUI2), while in the remaining two [102, 150] no considerations were given by the authors. The report TA443 referred to another study (full reference not provided), already used in a TA on sofosbuvir for treating chronic hepatitis C. One report (HST9) used EQ-5D utilities estimated in a previous study (full reference not provided) on the same condition (i.e., hereditary transthyretin amyloidosis), but with a different country value set. One report (TA491) addressing Waldenstrom’s macroglobulinaemia referred to two published studies [157, 158] on chronic lymphocytic leukaemia (which were not captured by our review, since we excluded cancer). In four cases (TA266, HST2, HST7, TA588), no details were given on the literature source used.

Overall, the association between study authors’ methodological considerations and manufacturers’ choices is not straightforward. In some cases, such as TA398, the use of EQ-5D is corroborated by several studies [49, 50, 59, 67] arguing the instrument is a valid and responsive instrument in cystic fibrosis. Similarly, in idiopathic pulmonary fibrosis, five (out of seven) available studies at the time of NICE assessment report (TA379) used EQ-5D and one [85] also reassured on its sensitivity in this condition. In other cases, such as HST2 and HST8, the manufacturers adopted EQ-5D despite the negative judgment reported by the literature, which showed that it did not correlate with pain measures in Mucopolysaccharidosis type IVa [86] or considered it as unable to capture specific issues in X-linked hypophosphatemia [80]. In IRDs, despite EQ-5D was shown to present significant ceiling effects and considered unsuitable for patients with eye conditions [70], the manufacturer (HST11) used both EQ-5D and HUI3, while the ERG suggested to refer to a previous study deriving TTO values from the general public. In these cases, the choice of using EQ-5D is more likely motivated by the willingness to comply with NICE’s recommendations than with the literature’s considerations. The first report on cystic fibrosis (TA266), in using the HUI3, might have followed some positive judgments on this instrument as reported by studies available at that time [50, 91, 125, 153], which highlighted its responsiveness, ease of understanding, and inclusion of relevant items, such as hearing and fertility, while the evidence on the use of EQ-5D was still scarce. Finally, there are few cases where the manufacturers’ methodological choices seem completely unrelated to the literature. For example, in HST4, a DCE with the public was presented, in spite of the availability of multiple studies using EQ-5D and suggestion from ERG to use values from the literature (unspecified).

## Discussion

### Synthesis of results

This study aimed to identify, examine and compare the approaches used in NICE technology appraisals and published literature on selected RDs in order to understand the level of agreement between the two and whether the manufacturers fully exploited the existing literature in developing their HTA submissions. A total of 22 TA or HST appraisals encompassing 19 different conditions were downloaded from NICE website. In a substantial number of submissions (10/22, 45.5%), the manufacturers used HSUVs from the literature, as the only approach or combined with primary utility data collected in clinical trials, to inform specific health states (e.g., lung transplantation in reports on cystic fibrosis, TA266 and TA398). However, in more than one third of submissions (8/22), preference-based instruments (EQ-5D or HUI2) were collected from patients during clinical studies. The comments of NICE committees on the approach adopted by manufacturers to obtain utilities for their models were heterogeneous; as expected, more appreciation was expressed when the manufacturers collected EQ-5D directly from patients during a clinical trial (which is the NICE’s recommended approach [9]). In few cases the ERG suggested a completely different approach to estimate HSUVs (e.g., in TA276, using literature values instead of mapping) while more often it recommended only minor adjustments to the manufacturer’s choices.

A total of 111 published studies were identified from the literature, of which one was counted twice because of addressed two different conditions in two separate article sections; therefore, data analysis was performed on 112 studies. One third were related to cystic fibrosis that, with a current prevalence of 11.1 per 100,000 [21], is one of the most common RDs. Almost one fourth were conducted in multiple countries, which is frequent in RDs due to high patients’ geographical dispersion [12]. More than two thirds were published in the last decade (2011–2020), which demonstrates a growing attention for QoL in RDs (and in general), and its incorporation into HTA appraisals [16, 19].

A total of 97 studies (86.6%) used at least one preference-based instrument to estimate HSUVs. Of the six main generic PROMs reported in the literature [8], all were used except for the Australian AQoL. Of them, the most frequently adopted (in 64.3% of studies, 72/112) was by far a EuroQol questionnaire (EQ-5D-3L, EQ-5D-5L or EQ-5D-Y), followed by HUI2/3 (14/112, 12.5%). The three-level version of EQ-5D (i.e., EQ-5D-3L) was first introduced by EuroQol in 1990 (www.euroqol.org), but never used in this review before 2000. The HUI, introduced in 2003, has been rarely but constantly adopted in the included studies since the beginning of the new millennium. The QWB, developed in the 1970s, was adopted by 11 studies (9.8%), all published before 2005, and subsequently likely replaced by more recent instruments. The 15D was used only by three Finnish studies [46, 47, 83], and the SF-6D by one study only [152] (but addressing two different RDs) and together with EQ-5D. The use of direct techniques was much less frequent; indeed, only 14 studies (12.5%) relied on these methodologies, of which 11 (9.8%) used SG. Finally, only three studies (2.7%) [48, 53, 101] performed a mapping exercise to obtain HSUVs from non-preference-based measures. This is not surprisingly, given the limitations related to the use of mapping in RDs emerged from our previous review [13].

In 11 studies (9.8%), parents (or other caregivers) were involved in proxy-reporting on patients’ status in addition to or in place of their children (especially if aged below 12). This review confirmed a growing consideration of caregivers’ and, in general, families’ well-being in research [16], since 12 studies (10.7%) interviewed the main carers about their own status, and also in HTA [15], as observed in three NICE appraisals (TA588, HST3, HST12) and related positive comments from ERG. The participants enrolled were less than 100 in 42% of studies, which is frequent in RDs where small-scale studies are usually conducted [12, 16, 19].

The degree of overlap between the methods adopted by manufacturers for their submission to NICE and published studies was quite limited and varied a lot according to the disease considered. In particular, the use of ‘vignettes’ valued by clinical experts and reliance on expert opinion to estimate HSUVs was much more frequent in manufacturers’ submissions than in the published literature (5/22, 22.7% vs. 9/112, 8.0%), where the great majority of studies (85.7%) surveyed patients directly to value their own status. It is worth noting that four (out of five) NICE technology appraisals reporting the use of vignettes were HST targeting ultra-RDs, in which it may be difficult to recruit patients. The reasons for diverging from the existing literature might be different. In some cases (e.g., HST6 and HST12), we realized that there were no published studies to refer to, while in others (e.g., HST1 and HST8) very few studies were available at the time of submission to NICE. Again, this situation was much more common for HST targeting ultra-rare diseases. In the submission related to IRDs (HST11), the company expressed a preference for HUI3 instead of EQ-5D, recommended by NICE and used by a previous study [70]), because the former contains a vision component while the latter is known to have poor validity in eye disorders [30]. In other reports, the patients’ characteristics, in terms of age or disease’s subtype, might be different from the samples enrolled in published studies, thus inducing manufacturers to perform a new data collection or embrace different approaches. For example, the eculizumab submission (HST1 [26]) also included paediatric patients (0–11 years), while the study retrieved in atypical haemolytic–uremic syndrome recruited only patients above 12 [103]. Similarly, burosumab (HST8 [24]) was authorized for children and adolescents, while the corresponding study on X-linked hypophosphatemia was focused on adults [80]; this might have incentivized the manufacturer to conduct an ad hoc vignette study where clinicians, instead of patients, completed the EQ-5D. However, it is difficult to identify a clear pattern in manufacturers’ choices, but in several cases, they seem unrelated to the literature. The choice of using EQ-5D, indeed, is more likely motivated by the willing to comply with NICE methods guidance [9]. In the appraisal reports reviewed, manufacturers rarely referred to (or commented on) the available literature on HSUVs. In few cases, the company admitted using literature data, because QoL or utility data were not (or not sufficiently) collected in clinical trials (HST7, TA606, TA443), or there were no suitable mapping algorithms to convert PROMs used in the clinical studies (e.g., Norfolk QOL-DN, PedsQL) to the EQ-5D or other utilities (HST3, HST9, HST11). In other cases (HST8, HST11), the manufacturers reported the lack of literature data (and/or mapping algorithms) and, therefore, the necessity to commission an ad hoc vignette study. Future research should adopt qualitative methods (e.g., focus groups, interviews) to investigate the deep reasons why manufacturers exploited (or not) the literature to inform their new drug submissions.

### Limitations

Our research has several limitations. First, in order to limit the number of studies to be included in the literature, we deliberately excluded those using secondary sources to derive HSUVs. Thus, we could not compare NICE technology appraisals and published studies in terms of the frequency in use of such an approach. Second, we only searched two databases (PubMed and ScHARRHUD), while using other sources (e.g., Embase) and checking the reference lists of the included studies could have provided additional results. Third, except for the pilot phase, the data extraction was done independently by a single author, while should be conducted by at least two people [159]. Fourth, we did not perform any quality assessment of the included studies; however, this task is considered optional by PRISMA-ScR (item 12) [22]. Fifth, although we considered studies published at least one year before the NICE report date to understand whether manufacturers fully exploited the literature, it is possible that they develop their economic analyses more than one year ahead of the submission to NICE, in which case they would not be able to consult and reference some of the studies used for comparing the approaches. Therefore, the comparison between NICE reports and the literature should be interpreted with caution and not be viewed as direct criticism of manufacturers, but rather as an encouragement for using different approaches or values existing in the literature in future submissions, given the availability of estimates and the methodological considerations discussed by authors.

### Key learnings

This review provided several insights into research and HTA. The critical issues highlighted in previous studies undertaken within the IMPACT-HTA project [12, 13, 19] and related to the general use of PROMs and the estimation of HSUVs in RDs were confirmed by the manuscripts reviewed, as well as their authors’ considerations. First, due to the low prevalence of RDs, the samples recruited in studies were generally small and might have affected the validity of results. In several cases, the ultra-rarity of condition (e.g., IRDs) hampered the enrolment of a representative patient sample and required the use of ‘vignettes’. The wide use of non-patient populations is a key issue in the estimation of HSUVs in RDs, since most patients are children or cognitively impaired, and cannot perform complex tasks, such as those required by direct techniques. Second, even in studies using simpler indirect approaches (e.g., EQ-5D), patients were often children who necessarily required parental support in order to fill in the required questionnaire to obtain HSUVs. The use of paediatric versions of instruments (e.g., EQ-5D-Y) can overcome the issue only partially, since they do not apply to mentally disabled or very young children.

Third, despite their advantages (i.e., simplicity, cheapness, comparability across different conditions), preference-based PROMs showed poor sensitivity in several RDs, due to their heterogeneity and peculiarities. For example, in conditions implying severe physical impairment (e.g., DMD), the EQ-5D does not consider mobility issues beyond walking, or do not include visual domains for eye conditions (e.g., limbal stem cell deficiency), unless adding the vision bolt-on. Moreover, EQ-5D also presented ceiling effects in mild disease. Fourth, patients’ geographical dispersion is likely to have required multi-country research studies and multi-lingual instruments, which inevitably imply more logistic and financial resources.

In this review, a variety of approaches were identified to estimate HSUVs. EQ-5D, which is currently the most frequently cited instrument (85%) in national pharmacoeconomic guidelines [160], was also the preferred instrument in the studies reviewed, with over 64% using it alone or in combination with other approaches. Similarly, 15 out of 22 (68.2%) manufacturers’ submissions presented EQ-5D utilities derived in different ways (i.e., from patients, from clinical experts using ‘vignettes’, by ‘mapping’ non-preference-based measures, or using values from the literature), in agreement with NICE guidelines indicating that EQ-5D can be sourced from the literature or by mapping, if not available in the clinical trials [9]. On the basis of methodological considerations extracted from published studies, EQ-5D is found to be an easy, valid and sensitive instrument in chronic lung diseases (i.e., cystic fibrosis and idiopathic pulmonary fibrosis), and also allows comparison with other conditions. However, it also includes irrelevant aspects (i.e., self-care) and present significant ceiling effects, especially in less severe health states. The instrument was presented as valid and appropriate also in hereditary transthyretin amyloidosis and hereditary angioedema. In DMD, instead, EQ-5D is valued as almost insensitive in capturing relevant disease’s changes, especially because does not consider any mobility aspects besides walking. The main EQ-5D limitations (i.e., poor sensitivity, ceiling effects) were also highlighted in other rare conditions (i.e., Fabry disease, IRD, mucopolysaccharidosis type Iva, X-linked hypophosphatemia). The NICE, on the other hand, acknowledged that the EQ-5D may not be the most appropriate instrument in some cases, and alternative QoL measures may be used provided that the lack of content validity for the EQ-5D is proven [9]. For example, in HST10, NICE’s committee considered that EQ-5D might not fully capture all the effects of autonomic neuropathy [29].

The comments about the other two frequently used preference-based PROMs (i.e., HUI2/3 and QWB) identified in the literature review (which were used by 12.5% and 9.8% of published studies, respectively) were heterogeneous. Similar to EQ-5D, HUI2/3 was found to perform well in rare lung diseases and include relevant dimensions (e.g., hearing, fertility) for cystic fibrosis patients, but not relevant enough for physically impaired patients, such as those affected by DMD and SMA. QWB was used by eleven studies all performed in chronic lung diseases (i.e., cystic fibrosis and idiopathic pulmonary fibrosis), where the evaluation of its performance and validity was mixed. In relation to direct techniques, it is worth mentioning that two studies [61, 148] stated that SG tends to over or underestimate HSUVs, depending on respondent’s willingness to accept the risk. Finally, the use of ‘vignettes’ valued by non-patient populations was often perceived as a ‘second-best’ approach justified by low prevalence of disease and consequent difficulties in recruiting patients [107]. Indeed, some studies [84, 138] highlighted the limitations of such an approach, mainly regarding standard descriptions of health states that do not capture the heterogeneous manifestations of diseases, development of vignettes in the absence of patient’s inputs, limited overlap between health state descriptions and the instrument (e.g., EQ-5D) used to value them, and difficulties for the general public to identify themselves in hypothetical RD patient descriptions.

## Conclusions

This is the first paper that analyses and compares the methods used to estimate HSUVs in NICE TA and HST appraisal of drugs with an EMA orphan designation with the published literature for the same targeted RDs. The study confirmed that EQ-5D is the preferred preference-based instrument, as recommended by NICE and other HTA authorities. The agreement on methodological choices between the two types of documents (i.e., NICE appraisals and published manuscripts) was only partial, since the former relied more often on ‘vignettes’ and clinical experts. Despite almost half of the appraisal reports reviewed referred to the literature (sometimes even without specifying the study referred to), more efforts should be made by manufacturers to exploit comprehensively the methods, results and considerations found in the published studies, which occasionally advises against using EQ-5D in selected RDs. At the same time, since methodological considerations retrieved from the existing literature are often inconsistent, future research could further investigate the most appropriate methods to estimate HSUVs for individual RDs and identify the ways to overcome the limitations of existing instruments. However, the wide use of EQ-5D in published research studies should provide some reassurance concerning the validity of this instrument at least in some RDs, such as those affecting respiratory system (i.e., cystic fibrosis and idiopathic pulmonary fibrosis). The use of condition-specific preference-based measures, which was very limited in our review, is also encouraged at least to determine their validity and responsiveness in comparison with the generic ones. Moreover, a deeper understanding of proxy-reporting for paediatric or cognitively impaired patients is required, to assess the validity of this frequently used approach in RDs, as well as the impact of including their carers’ QoL on HTA results.

## Supplementary Information

Below is the link to the electronic supplementary material.

Supplementary file1 (DOCX 15 KB)

Supplementary file2 (DOCX 84 KB)
