# Supplementary material for: The estimation of health state utility values in rare diseases: do the approaches in submissions for NICE technology appraisals reflect the existing literature? A scoping review
Source: Eur J Health Econ. 2022 Nov 5;24(7):1151–216. doi: 10.1007/s10198-022-01541-y (PMC10406664; doi:10.1007/s10198-022-01541-y)
Supplement: Supplementary file 1 — Supplementary file1 (DOCX 15 KB) [file 10198_2022_1541_MOESM1_ESM.docx]

**S1.** Full search strategy for PubMed.

1. ((Asfotase alfa)[Title/Abstract] OR (hypophosphatasia)[Title/Abstract]) AND ((health state utility values)[Title/Abstract] OR (utility values)[Title/Abstract] OR (health utilities)[Title/Abstract] OR (preference weights)[Title/Abstract] OR (index values)[Title/Abstract] OR QALYs[Title/Abstract] OR (cost-utility)[Title/Abstract] OR EQ-5D[Title/Abstract] OR EuroQol[Title/Abstract] OR HUI[Title/Abstract] OR (Health Utility Index)[Title/Abstract] OR QWB[Title/Abstract] OR SF-6D[Title/Abstract] OR 15D [Title/Abstract]).
2. ((Eculizumab)[Title/Abstract] OR (atypical haemolytic uraemic syndrome)[Title/Abstract]) AND ((health state utility values)[Title/Abstract] OR (utility values)[Title/Abstract] OR (health utilities)[Title/Abstract] OR (preference weights)[Title/Abstract] OR (index values)[Title/Abstract] OR QALYs[Title/Abstract] OR (cost-utility)[Title/Abstract] OR EQ-5D[Title/Abstract] OR EuroQol[Title/Abstract] OR HUI[Title/Abstract] OR (Health Utility Index)[Title/Abstract] OR QWB[Title/Abstract] OR SF-6D[Title/Abstract] OR 15D [Title/Abstract]).
3. ((Migalastat)[Title/Abstract] OR (Fabry disease)[Title/Abstract]) AND ((health state utility values)[Title/Abstract] OR (utility values)[Title/Abstract] OR (health utilities)[Title/Abstract] OR (preference weights)[Title/Abstract] OR (index values)[Title/Abstract] OR QALYs[Title/Abstract] OR (cost-utility)[Title/Abstract] OR EQ-5D[Title/Abstract] OR EuroQol[Title/Abstract] OR HUI[Title/Abstract] OR (Health Utility Index)[Title/Abstract] OR QWB[Title/Abstract] OR SF-6D[Title/Abstract] OR 15D [Title/Abstract]).
4. ((Inotersen)[Title/Abstract] OR (Patisiran)[Title/Abstract] OR (hereditary transthyretin amyloidosis)[Title/Abstract]) AND ((health state utility values)[Title/Abstract] OR (utility values)[Title/Abstract] OR (health utilities)[Title/Abstract] OR (preference weights)[Title/Abstract] OR (index values)[Title/Abstract] OR QALYs[Title/Abstract] OR (cost-utility)[Title/Abstract] OR EQ-5D[Title/Abstract] OR EuroQol[Title/Abstract] OR HUI[Title/Abstract] OR (Health Utility Index)[Title/Abstract] OR QWB[Title/Abstract] OR SF-6D[Title/Abstract] OR 15D [Title/Abstract]).
5. ((Voretigene)[Title/Abstract] OR (inherited retinal dystrophies)[Title/Abstract]) AND ((health state utility values)[Title/Abstract] OR (utility values)[Title/Abstract] OR (health utilities)[Title/Abstract] OR (preference weights)[Title/Abstract] OR (index values)[Title/Abstract] OR QALYs[Title/Abstract] OR (cost-utility)[Title/Abstract] OR EQ-5D[Title/Abstract] OR EuroQol[Title/Abstract] OR HUI[Title/Abstract] OR (Health Utility Index)[Title/Abstract] OR QWB[Title/Abstract] OR SF-6D[Title/Abstract] OR 15D [Title/Abstract]).
6. ((colistimethate sodium)[Title/Abstract] OR (tobramycin dry powders)[Title/Abstract] OR mannitol[Title/Abstract] OR lumacaftor[Title/Abstract] OR ivacaftor[Title/Abstract] OR (cystic fibrosis)[Title/Abstract]) AND ((health state utility values)[Title/Abstract] OR (utility values)[Title/Abstract] OR (health utilities)[Title/Abstract] OR (preference weights)[Title/Abstract] OR (index values)[Title/Abstract] OR QALYs[Title/Abstract] OR (cost-utility)[Title/Abstract] OR EQ-5D[Title/Abstract] OR EuroQol[Title/Abstract] OR HUI[Title/Abstract] OR (Health Utility Index)[Title/Abstract] OR QWB[Title/Abstract] OR SF-6D[Title/Abstract] OR 15D [Title/Abstract]).
7. ((Elosulfase alpha)[Title/Abstract] OR (mucopolysaccharidosis type IVa)[Title/Abstract]) AND ((health state utility values)[Title/Abstract] OR (utility values)[Title/Abstract] OR (health utilities)[Title/Abstract] OR (preference weights)[Title/Abstract] OR (index values)[Title/Abstract] OR QALYs[Title/Abstract] OR (cost-utility)[Title/Abstract] OR EQ-5D[Title/Abstract] OR EuroQol[Title/Abstract] OR HUI[Title/Abstract] OR (Health Utility Index)[Title/Abstract] OR QWB[Title/Abstract] OR SF-6D[Title/Abstract] OR 15D [Title/Abstract]).
8. ((Burosumab)[Title/Abstract] OR (X-linked hypophosphatemia)[Title/Abstract]) AND ((health state utility values)[Title/Abstract] OR (utility values)[Title/Abstract] OR (health utilities)[Title/Abstract] OR (preference weights)[Title/Abstract] OR (index values)[Title/Abstract] OR QALYs[Title/Abstract] OR (cost-utility)[Title/Abstract] OR EQ-5D[Title/Abstract] OR EuroQol[Title/Abstract] OR HUI[Title/Abstract] OR (Health Utility Index)[Title/Abstract] OR QWB[Title/Abstract] OR SF-6D[Title/Abstract] OR 15D [Title/Abstract]).
9. ((Strimvelis)[Title/Abstract] OR (adenosine deaminase deficiency)[Title/Abstract] OR (severe combined immunodeficiency)[Title/Abstract]) AND ((health state utility values)[Title/Abstract] OR (utility values)[Title/Abstract] OR (health utilities)[Title/Abstract] OR (preference weights)[Title/Abstract] OR (index values)[Title/Abstract] OR QALYs[Title/Abstract] OR (cost-utility)[Title/Abstract] OR EQ-5D[Title/Abstract] OR EuroQol[Title/Abstract] OR HUI[Title/Abstract] OR (Health Utility Index)[Title/Abstract] OR QWB[Title/Abstract] OR SF-6D[Title/Abstract] OR 15D [Title/Abstract]).
10. ((Nusinersen)[Title/Abstract] OR (spinal muscular atrophy)[Title/Abstract]) AND ((health state utility values)[Title/Abstract] OR (utility values)[Title/Abstract] OR (health utilities)[Title/Abstract] OR (preference weights)[Title/Abstract] OR (index values)[Title/Abstract] OR QALYs[Title/Abstract] OR (cost-utility)[Title/Abstract] OR EQ-5D[Title/Abstract] OR EuroQol[Title/Abstract] OR HUI[Title/Abstract] OR (Health Utility Index)[Title/Abstract] OR QWB[Title/Abstract] OR SF-6D[Title/Abstract] OR 15D [Title/Abstract]).
11. ((Mepolizumab)[Title/Abstract] OR (severe refractory eosinophilic asthma)[Title/Abstract]) AND ((health state utility values)[Title/Abstract] OR (utility values)[Title/Abstract] OR (health utilities)[Title/Abstract] OR (preference weights)[Title/Abstract] OR (index values)[Title/Abstract] OR QALYs[Title/Abstract] OR (cost-utility)[Title/Abstract] OR EQ-5D[Title/Abstract] OR EuroQol[Title/Abstract] OR HUI[Title/Abstract] OR (Health Utility Index)[Title/Abstract] OR QWB[Title/Abstract] OR SF-6D[Title/Abstract] OR 15D [Title/Abstract]).
12. ((Eliglustat)[Title/Abstract] OR (Gaucher disease)[Title/Abstract]) AND ((health state utility values)[Title/Abstract] OR (utility values)[Title/Abstract] OR (health utilities)[Title/Abstract] OR (preference weights)[Title/Abstract] OR (index values)[Title/Abstract] OR QALYs[Title/Abstract] OR (cost-utility)[Title/Abstract] OR EQ-5D[Title/Abstract] OR EuroQol[Title/Abstract] OR HUI[Title/Abstract] OR (Health Utility Index)[Title/Abstract] OR QWB[Title/Abstract] OR SF-6D[Title/Abstract] OR 15D [Title/Abstract]).
13. ((Holoclar)[Title/Abstract] OR (limbal stem cell deficiency)[Title/Abstract]) AND ((health state utility values)[Title/Abstract] OR (utility values)[Title/Abstract] OR (health utilities)[Title/Abstract] OR (preference weights)[Title/Abstract] OR (index values)[Title/Abstract] OR QALYs[Title/Abstract] OR (cost-utility)[Title/Abstract] OR EQ-5D[Title/Abstract] OR EuroQol[Title/Abstract] OR HUI[Title/Abstract] OR (Health Utility Index)[Title/Abstract] OR QWB[Title/Abstract] OR SF-6D[Title/Abstract] OR 15D [Title/Abstract]).
14. ((Lanadelumab)[Title/Abstract] OR (hereditary angioedema)[Title/Abstract]) AND ((health state utility values)[Title/Abstract] OR (utility values)[Title/Abstract] OR (health utilities)[Title/Abstract] OR (preference weights)[Title/Abstract] OR (index values)[Title/Abstract] OR QALYs[Title/Abstract] OR (cost-utility)[Title/Abstract] OR EQ-5D[Title/Abstract] OR EuroQol[Title/Abstract] OR HUI[Title/Abstract] OR (Health Utility Index)[Title/Abstract] OR QWB[Title/Abstract] OR SF-6D[Title/Abstract] OR 15D [Title/Abstract]).
15. ((Obeticholic acid)[Title/Abstract] OR (primary biliary cholangitis)[Title/Abstract]) AND ((health state utility values)[Title/Abstract] OR (utility values)[Title/Abstract] OR (health utilities)[Title/Abstract] OR (preference weights)[Title/Abstract] OR (index values)[Title/Abstract] OR QALYs[Title/Abstract] OR (cost-utility)[Title/Abstract] OR EQ-5D[Title/Abstract] OR EuroQol[Title/Abstract] OR HUI[Title/Abstract] OR (Health Utility Index)[Title/Abstract] OR QWB[Title/Abstract] OR SF-6D[Title/Abstract] OR 15D [Title/Abstract]).
16. ((Ibrutinib)[Title/Abstract] OR (Waldenstrom’s macroglobulinaemia)[Title/Abstract]) AND ((health state utility values)[Title/Abstract] OR (utility values)[Title/Abstract] OR (health utilities)[Title/Abstract] OR (preference weights)[Title/Abstract] OR (index values)[Title/Abstract] OR QALYs[Title/Abstract] OR (cost-utility)[Title/Abstract] OR EQ-5D[Title/Abstract] OR EuroQol[Title/Abstract] OR HUI[Title/Abstract] OR (Health Utility Index)[Title/Abstract] OR QWB[Title/Abstract] OR SF-6D[Title/Abstract] OR 15D [Title/Abstract]).
17. ((Nintedanib)[Title/Abstract] OR (idiopathic pulmonary fibrosis)[Title/Abstract]) AND ((health state utility values)[Title/Abstract] OR (utility values)[Title/Abstract] OR (health utilities)[Title/Abstract] OR (preference weights)[Title/Abstract] OR (index values)[Title/Abstract] OR QALYs[Title/Abstract] OR (cost-utility)[Title/Abstract] OR EQ-5D[Title/Abstract] OR EuroQol[Title/Abstract] OR HUI[Title/Abstract] OR (Health Utility Index)[Title/Abstract] OR QWB[Title/Abstract] OR SF-6D[Title/Abstract] OR 15D [Title/Abstract]).
18. ((Ataluren)[Title/Abstract] OR (Duchenne muscular dystrophy)[Title/Abstract]) AND ((health state utility values)[Title/Abstract] OR (utility values)[Title/Abstract] OR (health utilities)[Title/Abstract] OR (preference weights)[Title/Abstract] OR (index values)[Title/Abstract] OR QALYs[Title/Abstract] OR (cost-utility)[Title/Abstract] OR EQ-5D[Title/Abstract] OR EuroQol[Title/Abstract] OR HUI[Title/Abstract] OR (Health Utility Index)[Title/Abstract] OR QWB[Title/Abstract] OR SF-6D[Title/Abstract] OR 15D [Title/Abstract]).
19. ((Cerliponase alfa)[Title/Abstract] OR (Neuronal ceroid lipofuscinosis)[Title/Abstract]) AND ((health state utility values)[Title/Abstract] OR (utility values)[Title/Abstract] OR (health utilities)[Title/Abstract] OR (preference weights)[Title/Abstract] OR (index values)[Title/Abstract] OR QALYs[Title/Abstract] OR (cost-utility)[Title/Abstract] OR EQ-5D[Title/Abstract] OR EuroQol[Title/Abstract] OR HUI[Title/Abstract] OR (Health Utility Index)[Title/Abstract] OR QWB[Title/Abstract] OR SF-6D[Title/Abstract] OR 15D [Title/Abstract]).
